# miR-1 sustains muscle physiology by controlling V-ATPase complex assembly

**DOI:** 10.1126/sciadv.abh1434

**Published:** 2021-10-15

**Authors:** Paula Gutiérrez-Pérez, Emilio M. Santillán, Thomas Lendl, Jingkui Wang, Anna Schrempf, Thomas L. Steinacker, Mila Asparuhova, Marlene Brandstetter, David Haselbach, Luisa Cochella

**Affiliations:** 1 Research Institute of Molecular Pathology (IMP), Vienna BioCenter (VBC), 1030 Vienna, Austria.; 2 Vienna BioCenter PhD Program, Doctoral School of the University of Vienna and Medical University of Vienna, 1030 Vienna, Austria.; 3 Research Center for Molecular Medicine of the Austrian Academy of Sciences, 1090 Vienna, Austria.; 4 Sir William Dunn School of Pathology, University of Oxford, Oxford OX1 3RE, UK.; 5 Electron Microscopy Facility, Vienna BioCenter Core Facilities GmbH, Vienna, Austria.

## Abstract

Muscle function requires unique structural and metabolic adaptations that can render muscle cells selectively vulnerable, with mutations in some ubiquitously expressed genes causing myopathies but sparing other tissues. We uncovered a muscle cell vulnerability by studying miR-1, a deeply conserved, muscle-specific microRNA whose ablation causes various muscle defects. Using *Caenorhabditis elegans*, we found that miR-1 represses multiple subunits of the ubiquitous vacuolar adenosine triphosphatase (V-ATPase) complex, which is essential for internal compartment acidification and metabolic signaling. V-ATPase subunits are predicted miR-1 targets in animals ranging from *C. elegans* to humans, and we experimentally validated this in *Drosophila*. Unexpectedly, up-regulation of V-ATPase subunits upon miR-1 deletion causes reduced V-ATPase function due to defects in complex assembly. These results reveal V-ATPase assembly as a conserved muscle cell vulnerability and support a previously unknown role for microRNAs in the regulation of protein complexes.

## Teaser

A deeply conserved miR-1–dependent regulon of mitochondria and lysosome function is essential for muscle physiology.

## INTRODUCTION

Skeletal and cardiac muscle cells present complex intracellular and membrane structures, with large fractions of their cytoplasm dedicated to the contractile apparatus. In addition, they display unique metabolic adaptations to support their energy demands. Although these features are crucial for force generation and metabolic homeostasis, they also put constraints on the function of many organelles and on the protein and membrane trafficking machineries. Notably, several myopathies are caused by defects in mitochondria function, metabolic pathways, or membrane organization (*1*, *2*), suggesting that muscle cells are particularly vulnerable to impairments in these processes.

Cell-specific repressors of gene expression can provide insight into genetic pathways and cellular processes that a specialized cell may need to control in a particular manner. MicroRNAs (miRNAs) are posttranscriptional repressors, many of which are expressed with high cell-type specificity. miR-1 is specifically expressed in muscle and is one of only 32 miRNAs conserved throughout Bilateria (*3*). Its unique degree of conservation extends from its sequence and specificity of expression, to its profound impact on muscle development and physiology. Specifically, miR-1 is necessary for myoblast differentiation and survival as well as for sarcomere structure in most animals (*4*–*9*). Moreover, it is required for energy homeostasis and general physiology of muscle cells (*10*, *11*). Loss of miR-1 causes perinatal lethality in mice due to heart defects and larval lethality in *Drosophila* (*5*–*7*). Despite its critical role, the mechanism of action of this ancient, muscle-specific repressor is still poorly understood. Several different, nonconserved targets have been proposed to explain parts of its function [reviewed in (*12*)], a fact that is difficult to reconcile with the extreme conservation of miR-1. We set out to dissect the mechanism of action of miR-1 to gain insight into muscle-specific regulatory requirements.

Notably, miR-1 has predicted binding sites in transcripts encoding many of the 15 subunits that form the vacuolar adenosine triphosphatase (V-ATPase) complex in worms, flies, fish, mice, and humans (fig. S1) (*13*–*15*). This degree of target conservation is extremely rare across miRNAs, yet the functional link between miR-1 and the V-ATPase is completely unexplored. The V-ATPase complex drives the acidification of intracellular compartments, most notably lysosomes, and is thus essential for lysosomal degradation and autophagy. Beyond this function in catabolism, the V-ATPase is necessary for nutrient signaling and activation of mTORC and AMPK signaling (*16*–*18*). Given its central role in metabolism and cellular homeostasis (*19*, *20*), we hypothesized that V-ATPase subunits could constitute a conserved regulon that accounts for the muscle cell defects observed in miR-1 mutant animals.

## RESULTS

**Fig. 1.**
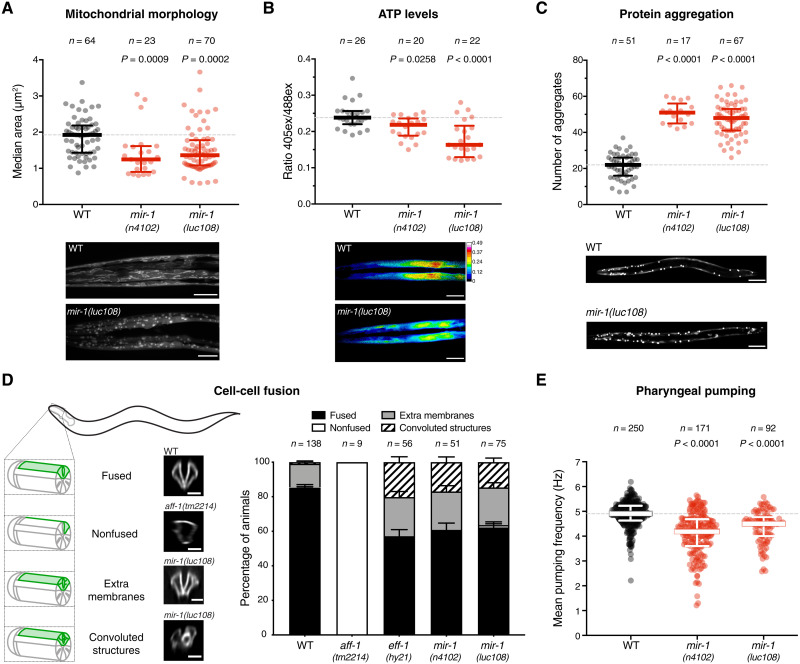
Absence of miR-1 causes diverse muscle defects in *C. elegans.* (**A**) Live L1 larvae of the indicated genotypes, expressing a mitochondria-localized GFP reporter in body-wall muscles, were imaged by confocal microscopy. Automated analysis (see Materials and Methods) was used to extract median mitochondria area (and other parameters in fig. S2D). *mir-1* mutant animals have smaller mitochondria in muscle. *P* values by Kruskal-Wallis test are indicated. Representative images are shown. Scale bars, 10 μm. WT, wild type. (**B**) Live L1 larvae expressing a ratiometric ATP sensor were imaged by confocal microscopy. Automated analysis (see Materials and Methods) was used to extract the emission ratio at 405- and 488-nm excitation (ex) wavelengths. *Mir-1(n4102* and *luc108)* animals have reduced ATP concentration in muscle. *P* values by Kruskal-Wallis test are indicated. Representative images are shown. Scale bars, 10 μm. (**C**) L4 animals of the indicated genotypes expressing the aggregation-prone Q40::YFP reporter were imaged, and the number of aggregates per animal was counted. *Mir-1* mutant animals have more aggregates. *P* values [one-way analysis of variance (ANOVA)] are indicated. Representative images are shown. Scale bars, 50 μm. (**D**) Left: Schematic representation of the cell-cell fusion assay: pm3DL and R fusion is assessed by the expression of a pm3DR-specific membrane marker (green). If fusion occurs, then the whole pm3D outline is labeled (fused), whereas if not, only pm3DR remains labeled (nonfused). If membrane trafficking is impaired or fusion is not completely achieved, then extra membranes or convoluted structures are observed. Representative images are shown. Scale bars, 20 μm. Right: Quantification of the cell-cell fusion events. *P* value (χ^2^) <0.0001. (**E**) Pumping frequencies of wild-type and *mir-1(n4102* and *luc108)* animals were extracted from electropharyngeograms (EPGs) conducted with the ScreenChip System in the presence of 10 mM serotonin. *Mir-1* mutant animals have reduced pumping frequency. *P* values by Kruskal-Wallis test are indicated. *n* is the number of animals analyzed.

To test this, we took advantage of *Caenorhabditis elegans* genetics and the fact that miR-1–deficient worms are viable in the laboratory, albeit with a few characterized muscle cell defects (*21*, *22*). We first characterized structural and metabolic properties of miR-1–deficient muscle cells, to establish quantitative assays that could be used for functional genetics. We visualized the sarcomeric structure of the body-wall muscle by electron microscopy, immunostaining, and live imaging of transgenic animals carrying a myosin heavy chain (MYO-3) fused to green fluorescent protein (GFP), using two different mutant alleles of *mir-1* (fig. S2A). In contrast to other animals (*6*, *8*, *10*), we observed no obvious loss of sarcomere integrity (fig. S2, B and C). However, similar to mouse skeletal muscle, where loss of miR-1 causes disruption of mitochondrial structure and function (*11*, *23*), we found that miR-1–deficient L1 larvae displayed significant fragmentation of the otherwise extensive tubular mitochondrial network in the body-wall muscle (Fig. 1A and fig. S2D). This morphological defect was accompanied by reduced ATP levels in the body-wall muscle, measured with a fluorescent ATP sensor (Fig. 1B). Moreover, using an aggregation prone, polyglutamine–yellow fluorescent protein (YFP) reporter expressed in the cytoplasm of body-wall muscles, we observed an increase in the number of aggregates in the absence of miR-1 (Fig. 1C). This has also recently been reported in an independent study (*22*). The number of aberrant aggregates was significantly reduced by transgenically restoring miR-1 expression (fig. S2E).

In mammals, loss of miR-1 results in decreased cell-cell fusion, impairing myotube formation (*24*). While body-wall muscles do not fuse in *C. elegans*, pharyngeal muscle cells do (*25*); we thus developed an assay to monitor the fusion of two specific pharyngeal muscle cells, pm3DL and pm3DR (Fig. 1D). We monitored fusion in animals defective for miR-1 or for the known *C. elegans* cell-cell fusogens, EFF-1 and AFF-1 (*26*). pm3DL/R fusion was completely abolished in *aff-1* mutants, confirming that AFF-1 is the fusogen that initiates pm3D fusion (*27*). Animals deficient for *eff-1* or for miR-1 looked indistinguishable from each other but different from *aff-1* mutants: The pm3D cells appear to fuse but display ectopic labeled membranes that, in some cases, formed highly convoluted structures (Fig. 1D), indicating that EFF-1 also plays a role in these cells. This is reminiscent of the complex membrane structures that accumulate in unfused hypodermal cells in the context of a semipermissive allele of *eff-1* (*28*) and also of other membrane abnormalities attributed to defects in intracellular scission and fission events in *eff-1* and *aff-1* mutants (*29*, *30*). Together, this suggests that miR-1–deficient animals may have decreased expression or activity of EFF-1. Notably, trafficking and recycling of EFF-1 are altered upon V-ATPase loss of function in the *C. elegans* hypodermis (*31*, *32*), suggesting a potential link between miR-1 function and the observed membrane-related phenotypes.

We also asked whether the cellular defects observed in miR-1 mutants translated into locomotion or pharyngeal pumping defects. We did not observe significant differences in posture or speed of locomotion between wild-type and miR-1–deficient L4/young adult animals, but we did measure a decrease in pharyngeal pumping frequency in miR-1 mutants (Fig. 1E). This was due to extended duration of each pumping cycle, specifically during the muscle relaxation phase (fig. S2, F and G). Overall, *C. elegans* muscle cells lacking miR-1 display a broad range of cellular defects that could have a common origin in a disrupted mitochondrial-lysosomal axis, affecting metabolism, proteostasis, and membrane trafficking. The similarity in cellular phenotypes across multiple, diverse animals indicates a deeply conserved cellular function for this miRNA.

**Fig. 2.**
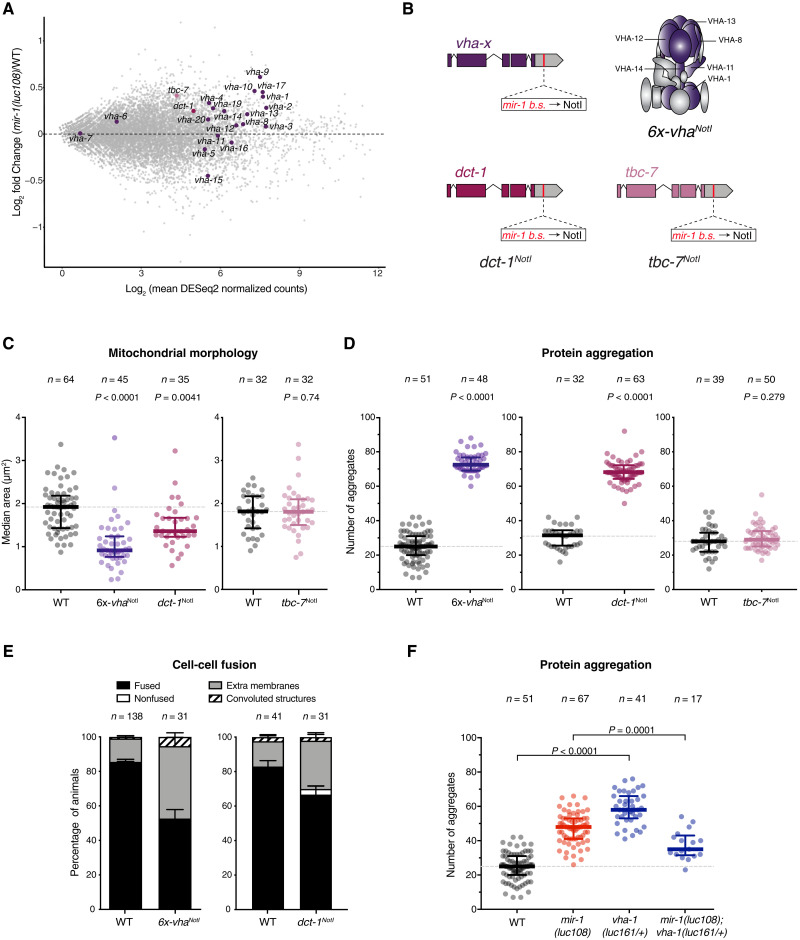
Direct repression of V-ATPase subunits and DCT-1 by miR-1 is necessary for muscle homeostasis. (**A**) MA plot for differentially expressed genes in sorted muscle cells from wild-type and *mir-1(luc108)* 1.5-fold embryos. All *vha* subunits, *dct-1*, and *tbc-7* are highlighted. (**B**) Schematic representing the design of *6x-vha*^NotI^, *dct-1*^NotI^, and *tbc-7*^NotI^ mutant strain. The *mir-1* binding sites in the 3′UTRs of *vha-1*, *vha-8*, *vha-11*, *vha-12*, *vha-13*, and *vha-14* (*6x-vha*^NotI^), or *dct-1*, or *tbc-7* were replaced by Not I restriction sites. (**C**) Median mitochondria area, extracted by automated analysis of images as in Fig. 1A, is significantly reduced in both *6x-vha*^NotI^ and *dct-1*^NotI^ (left) but not in *tbc-7*^NotI^ L1 larvae (right). *P* values by Kruskal-Wallis test (left) and unpaired *t* test (right) are indicated. Wild-type dataset in left graph is replotted from Fig. 1A. (**D**) Number of aggregates per animal measured as in Fig. 1C; *6x-vha*^NotI^ (left) and *dct-1*^NotI^ (middle) L4 animals show impaired proteostasis, but not *tbc-7*^NotI^ mutants (right). *P* values (unpaired *t* test) are shown. (**E**) Quantification of the different fusion outcomes as in Fig. 1D; *6x-vha*^NotI^ L1 animals (left) display both internal and external membranes in pharyngeal muscle cells, whereas *dct-1*^NotI^ L1 animals (right) only have a slight increase in internal membranes. *P* value (χ^2^) <0.0001 and 0.0345, respectively. Wild-type dataset (left) is replotted from Fig. 1D. (**F**) Reduction in *vha-1* levels rescues *mir-1(luc108)* proteostasis defects, as indicated by a significant decrease in the number of Q40::YFP aggregates per animal. *P* values (one-way ANOVA) are shown. Wild-type and *mir-1(luc108)* datasets are replotted from Fig. 1C. *n* refers to number of animals analyzed.

To evaluate the molecular changes caused by miR-1 loss in muscle, we performed transcriptome profiling of sorted muscle cells from wild-type and *mir-1* mutant embryos at the 1.5-fold stage. Gene category analysis of these deregulated transcripts revealed that up-regulated ones (280 genes) are enriched for stress related genes, whereas down-regulated ones (115 genes) are enriched for muscle-specific and extracellular matrix annotations (*33*), including many components of the contractile machinery (fig. S3, A to C, and data file S1). Among the genes up-regulated in the absence of miR-1, 22 are predicted miR-1 targets by TargetScan, including eight V-ATPase–encoding transcripts (*vha*); five genes from the down-regulated set were also predicted targets. A global gene set enrichment analysis revealed a modest enrichment of predicted miR-1 targets among up-regulated genes (fig. S3D). This enrichment was stronger when focused specifically on the V-ATPase–encoding transcripts (*vha*), with the majority of the *vhas* being up-regulated between 15 and 50% in the absence of miR-1 (Fig. 2A, fig. S3E, and data file S1). Similar levels of up-regulation of the *vhas* transcripts were also detected by reverse transcription followed by quantitative polymerase chain reaction (RT-qPCR) on whole L1 larvae (fig. S3F).

In addition to the V-ATPase subunits, two other predicted targets of miR-1, *dct-1* and *tbc-7*, caught our attention as they are also up-regulated in miR-1–deficient muscles (Fig. 2A and fig. S3F) and show target site conservation (fig. S1A). Moreover, *tbc-7* was recently reported as a functional target of miR-1 (*22*). DCT-1/BNIP3 regulates mitophagy and preserves mitochondria turnover and cellular homeostasis under stress conditions or aging (*34*–*36*). TBC-7/TBC1D15 is a Rab guanosine triphosphatase–activating protein that regulates lysosomal morphology and fusion between late endosomes and lysosomes (*37*). DCT-1/BNIP3 had not been previously linked to miR-1 function, but TBC-7/TBC1D15 is derepressed in the absence of miR-1, and forced overexpression of TBC-7 leads to impaired autophagy and proteotoxic stress (*22*). We therefore set out to test whether these targets are also part of the conserved miR-1 regulatory axis.

We first assessed whether miR-1 directly represses the *vha* genes, *dct-1* and/or *tbc-7*, by using CRISPR-Cas9 to mutate the predicted miR-1 binding sites in the 3′ untranslated regions (3′UTRs) of *dct-1* (*dct-1^NotI^*), *tbc-7 (tbc-7^NotI^)*, or six *vha* genes simultaneously (*vha-1*, *vha-8*, *vha-11*, *vha-12*, *vha-13*, and *vha-14; 6x-vha^NotI^*). The selected *vha* genes have conserved predicted miR-1 binding sites and constitute different parts of the complex (the V_0_ membrane ring, the V_1_ head, or the V_1_ stalk that bridges both parts) (Fig. 2B and figs. S1 and S4A). Because of the challenges of sorting muscle cells, we measured transcript levels by RT-qPCR on whole larvae. While this limited the sensitivity of the assay, we still observed that the levels of all six *vha* transcripts in the *6x-vha^NotI^* mutant strain were increased relative to wild type in starved animals and mirrored those in miR-1–deficient animals (fig. S3F). For fed animals, we observed mostly the same trends but with higher variability. This indicates that miR-1 is a direct repressor of multiple *vha* genes (fig. S3F). The same trends were observed for *dct-1* and *tbc-7* in *dct-1^NotI^* and *tbc-7^NotI^* mutant strains, respectively (fig. S3F).

If transcripts encoding V-ATPase subunits, DCT-1, and/or TBC-7 are functionally relevant targets of miR-1, removal of the miR-1 binding sites from their 3′UTRs should cause similar defects to the loss of miR-1 itself. We thus assessed mitochondrial morphology, protein aggregation, and pharyngeal muscle fusion in the *6x-vha^NotI^* and *dct-1^NotI^* strains (Fig. 2, C to E), and the mitochondria- and proteostasis-related phenotypes in the *tbc-7^NotI^* strain (Fig. 2, C and D). All were in otherwise wild-type backgrounds in which miR-1 is present and can regulate all other putative targets. Derepression of the *vha* transcripts or of *dct-1*, but not of *tbc-7*, was sufficient to cause the same mitochondrial and proteostasis defects that we observed in *mir-1* mutant animals (Fig. 2, C and D). In contrast, the defects in pharyngeal muscle cell fusion observed in the absence of miR-1 were phenocopied by derepression of the *vha* transcripts but not of *dct-1* alone, suggesting a specific contribution of the V-ATPase to this aspect of miR-1 function (Fig. 2E). Providing additional support for the functional connection between the V-ATPase and miR-1, removing one genomic copy of *vha-1* reduced the number of protein aggregates in *mir-1* mutant animals (Fig. 2F).

These results indicate that both the V-ATPase and DCT-1/BNIP3 are functional targets of miR-1. Derepression of one or the other is sufficient to cause similar observed phenotypes, suggesting that they affect common cellular processes in such a way that deregulation of either one has a negative impact on both protein and mitochondria homeostasis. The simultaneous derepression of both *vhas* and *dct-1* did not enhance either defect (fig. S4, D and E). This is consistent with both targets acting in the same pathway, although it could also mean that the observed defects cannot be further enhanced. These observations highlight the need for miR-1 repression of multiple targets simultaneously to maintain cellular homeostasis. In contrast to *dct-1* and the *vhas*, the level of *tbc-7* derepression in the absence of miR-1-mediated regulation is not sufficient to cause the measured cellular defects, although forced overexpression of *tbc-7* had been reported to induce protein aggregation in *C. elegans* muscle (*22*). Together, these experiments establish a miR-1–dependent regulon of lysosome and mitochondria function, composed primarily of the V-ATPase and DCT-1, that accounts for the critical role of this miRNA in muscle physiology. Components of this regulon are also predicted targets in *Drosophila* and in multiple vertebrate species [TargetScan (*15*)].

Our genetic analysis indicates that the moderate up-regulation of *dct-1* or the tested *vha* transcripts is sufficient to cause pleiotropic muscle cell defects. Increased target levels could, in principle, result in gain-of-function or hyperactivity effects, which are thought to account for most overexpression phenotypes (*38*). Alternatively, increased expression could result in loss-of-function effects due to competitive or dominant-negative interactions (*39*).

In the case of DCT-1, hyperactivity is a likely cause for the defects observed upon its derepression: The mitochondrial fragmentation and protein aggregation that we measured mirror defects that had been observed upon BNIP3 overexpression, but not upon BNIP3 loss of function, in mammalian cells (*34*, *35*). However, this will require further exploration, given that knockdown of *dct-1* also causes mitochondrial fragmentation in *C. elegans* (*36*).

In the case of the V-ATPase complex, it was unclear whether derepression of its subunits caused a gain or a loss of function. On the one hand, increased expression of some individual subunits results in gain-of-function effects in diverse types of cancer, by causing increased localization of the V-ATPase complex to the plasma membrane and therefore acidification of the extracellular environment and other cellular defects (*19*). In plants, overexpression of individual subunits leads to increased V-ATPase activity and resistance to multiple stressors (*40*). On the other hand, decreased activity of the V-ATPase complex is known to cause impaired lysosomal degradation and autophagy, protein aggregation, and defects in metabolic signaling and mitochondrial morphology (*41*–*45*), which could explain the protein and mitochondria homeostasis defects that we observed upon derepression of multiple *vha* transcripts.

**Fig. 3.**
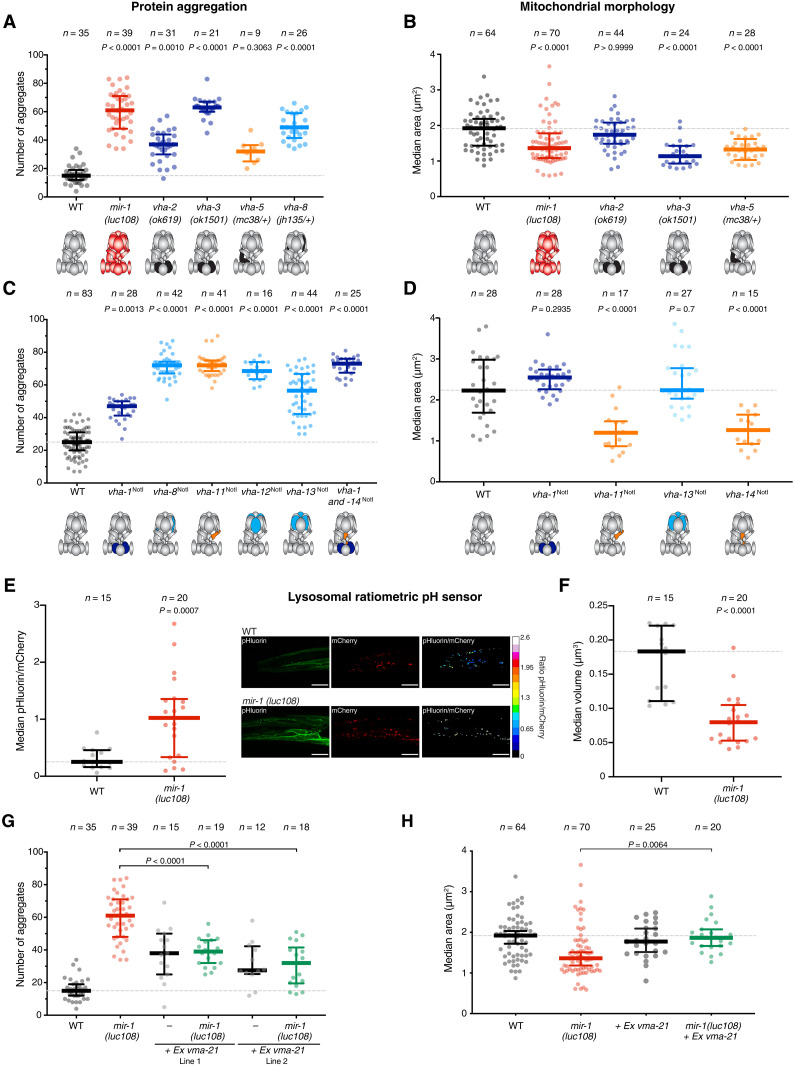
Loss of miR-1 results in decreased function of the V-ATPase complex due to assembly defects. (**A** and **B**) V-ATPase loss of function mutations in *vha-2*, *vha-3*, *vha-5*, or *vha-8* result in increased protein aggregation (A) and mitochondria fragmentation (B). *P* values by Kruskal-Wallis test are indicated. V-ATPase complex schematics are represented below, where black represents loss of function of each particular subunit. (**C**) Derepression of single subunits, by miR-1 binding site mutation, is sufficient to cause proteostasis defects. *P* values by Kruskal-Wallis test are indicated. V-ATPase complex schematics are represented below, where dark blue represents a membrane-integral subunit, orange a subunit of the connector stalk, and light blue a subunit involved in the hydrolysis of ATP. (**D**) Derepression of *vha-11* and *vha-14* alone, but not of *vha-1* or *vha-13*, is sufficient to cause mitochondrial network fragmentation. *P* values by Kruskal-Wallis test are indicated. (**E**) Live L1 larvae expressing a ratiometric pH sensor fused to LAMP1 were imaged by confocal microscopy. Automated analysis (see Materials and Methods) was used to extract the ratio of the superecliptic pHluorin to mCherry fluorescence. *Mir-1(luc108)* animals have a higher lysosomal pH, as indicated by a higher pHluorin/mCherry ratio. Each dot represents an animal (*n* = 15 to 20), and 30 to 50 lysosomes were measured per animal to extract the median ratio. *P* values by unpaired *t* test are indicated. Representative images are shown. Scale bars, 10 μm. (**F**) Lysosomal volume was extracted from the same images used for the analysis in (E). *mir-1(luc108)* animals have smaller lysosomes. *P* values by unpaired *t* test are indicated. (**G**) Overexpression of VMA-21 rescues miR-1 proteostasis defects. Two independent extrachromosomal lines are shown. Wild-type and *mir-1(luc108)* datasets are replotted from (A). *P* values by one-way ANOVA are shown. (**H**) Overexpression of VMA-21 rescues miR-1 mitochondrial network fragmentation. Wild-type and *mir-1(luc108)* datasets in (B) and (F) are replotted from Fig. 1A. *P* values by Kruskal-Wallis are shown.

To discern whether loss of miR-1 regulation causes a gain or a loss of function of the V-ATPase, we first asked whether reducing the level of V-ATPase subunits results in the same muscle-related defects as their up-regulation. Homozygous deletion of subunits with redundant counterparts (*vha-2/3*) or heterozygous mutation of unique subunits (*vha-1*, *vha-5*, and *vha-8*) had no impact on fertility or viability but caused significant increases in mitochondrial fragmentation and protein aggregation (Figs. 2F and 3, A and B), phenocopying both miR-1 mutant and *6x-vha^NotI^* animals. We further asked how deregulation of individual subunits affects mitochondrial structure and proteostasis in muscle. We reasoned that gain of function of the V-ATPase complex could occur if there was one or a subset of limiting subunits and their up-regulation enabled formation of more complexes, similar to what has been observed for proteasome assembly in yeast (*46*). In this scenario, only the up-regulation of the one or of all the limiting subunits simultaneously would lead to formation of more V-ATPase, but up-regulation of any other individual subunit would not. On the other hand, loss-of-function effects from protein overexpression arise from abnormal stoichiometry, which could lead to aberrant complex formation (*47*). In this scenario, up-regulation of multiple individual subunits could lead to similar defects. We found that removing the miR-1 binding sites from different individual subunits caused similar extent of Q40 protein aggregation as loss of miR-1 (Fig. 3C). Also, derepression of *vha-11* or *vha-14* alone resulted in mitochondrial fragmentation (Fig. 3D). These results are not consistent with V-ATPase gain of function and rather support the model in which up-regulation of V-ATPase subunits upon loss of miR-1 causes loss-of-function of the V-ATPase.

The main role of the V-ATPase complex is the acidification of internal compartments, most notably lysosomes. Thus, to further test the loss-of-function model, we measured lysosomal pH using a genetically encoded ratiometric pH sensor fused to the lysosomal protein LAMP1 (*48*). The sensor was expressed exclusively in the body-wall muscles of wild-type and miR-1–deficient *C. elegans.* Supporting the reduced activity of the V-ATPase complex, we observed a significant increase in lysosomal pH in miR-1 mutant animals (higher pHluorin/mCherry ratio) (Fig. 3E). This was accompanied by a significant decrease in lysosomal volume, consistent with altered lysosomal physiology (Fig. 3F).

Intriguingly, when manipulating individual subunits, we observed the strongest cellular defects upon deregulation of components of the stalk that couples the V_0_ and V_1_ subcomplexes (Fig. 3, C and D) (*49*). This led us to hypothesize that derepression of one or more subunits could disrupt the correct assembly of this intricate 15-subunit complex (*49*, *50*), ultimately resulting in reduced V-ATPase activity. To test this hypothesis, we attempted to rescue the muscle cell defects by restoring the assembly process in two ways. First, assembly of the V-ATPase requires dedicated chaperones in the endoplasmic reticulum (ER) (*50*). One of these chaperones, VMA21, is necessary in stoichiometric amounts to assist V_0_ assembly in the ER and its shuttling to the Golgi, where the V_0_ and the peripheral V_1_ subcomplexes come together (*51*). Notably, overexpression of the homolog of VMA21 in *C. elegans*, R07E5.7 (*52*), alleviated the defects observed in miR-1–deficient animals, significantly decreasing the number of cytoplasmic aggregates and the mitochondria fragmentation (Fig. 3, G and H). Highlighting the specificity of this rescue to the V-ATPase deregulation, overexpression of VMA21 did not rescue the same defects observed in the *dct-1^NotI^* mutant strain (fig. S5A). Second, we exploited the fact that glucose or amino acid deprivation has been shown to promote association of the V_0_ and V_1_ subcomplexes in mammalian cells (*53*, *54*). We thus asked whether starvation of L1 larvae had a beneficial effect on miR-1 mutant animals. Consistent with an improvement in V-ATPase function, the mitochondria morphology of starved miR-1–deficient animals was similar to the wild type (fig. S5B). In contrast, starvation was unable to rescue mitochondria fragmentation in animals in which V-ATPase loss of function was due to genomic deletion of a subunit (fig. S5B). Together, these observations suggest a role for miR-1 in enabling correct assembly of this crucial multisubunit complex in muscle cells, by regulating V-ATPase subunit production with the right levels, dynamics, and/or stoichiometry.

**Fig. 4.**
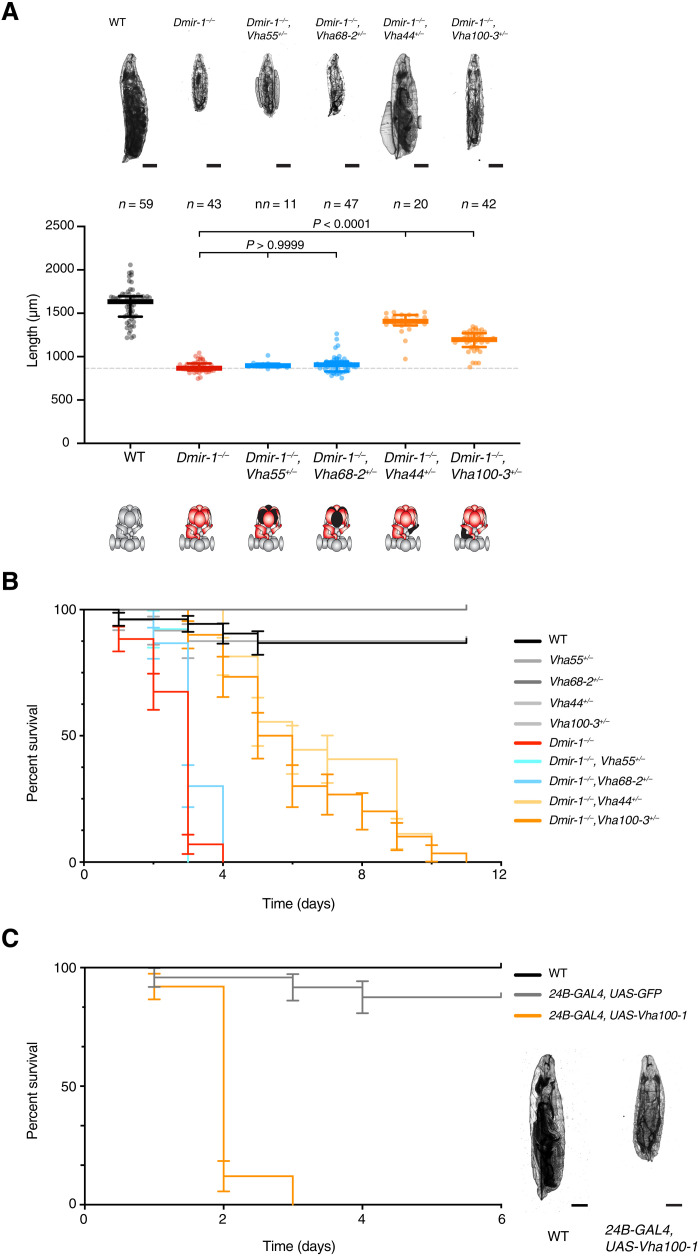
The miR-1/V-ATPase functional interaction is conserved in *Drosophila.* (**A**) Reduction in VHA100-3 or VHA44 levels, but not of VHA55 or VHA68-2, rescues *Dmir-1(0)* morphological defects. Top: Representative pictures of 24-hour posthatching larvae are shown. Scale bars, 200 μm. Bottom: Quantification of whole-body length. *n* represents number of animals analyzed. *P* values by Kruskal-Wallis test are indicated. (**B**) Reduction in VHA100-3 or VHA44 levels prolongs *Dmir-1(0)* life. Curves and error bars represent the average and SD of three independent biological replicates, consisting of 10 *Drosophila* larvae each. (**C**) Muscle-specific overexpression of VHA100-1 causes larval lethality 2 days after hatching. Curves and error bars represent the average and SD of three independent biological replicates, consisting of five to eight *Drosophila* larvae each. Representative pictures of larvae 24 hours after hatching are shown. Scale bars, 200 μm.

Last, we set out to test whether the remarkable conservation of the miR-1 sequence and the presence of predicted miR-1 binding sites in multiple V-ATPase subunits also reflects functional conservation. To this end, we analyzed *Drosophila melanogaster*, estimated to have diverged from nematodes ~600 million years ago. Flies carrying a deletion of miR-1 have various muscle defects that result in growth arrest as second instar larvae and lethality within 2 to 4 days of hatching, and these defects were rescued by a transgene expressing miR-1 in muscle (*6*). We first quantified the levels of various transcripts encoding V-ATPase subunits in first instar larvae and found that multiple *Vhas* were up-regulated in the same deletion allele of miR-1 used in (*6*) (fig. S6A). To test the functional connection between miR-1 and the V-ATPase complex, we asked whether reducing the levels of targeted subunits that are important for assembly of the V_0_ and V_1_ rings (*49*) might rescue the larval growth arrest. Akin to what we observed in *C. elegans*, deletion of one genomic copy of *Vha44* or of *Vha100-3* rescued the growth defect of miR-1–deficient larvae (Fig. 4A). In contrast, reducing the level of two other miR-1–targeted subunits that form the peripheral V_1_ ring (*Vha55* and *Vha68-2*) did not significantly rescue the growth defect (Fig. 4A). This is consistent with our observations in *C. elegans* that point to loss of miR-1 causing a V-ATPase assembly defect, primarily at the level of coupling the V_0_ and V_1_ subcomplexes. Concomitant with the increased growth, reducing the level of *Vha100-3* also significantly extended the survival of miR-1–deficient larvae (Fig. 4B), although these larvae did not pupate and reach adulthood. Conversely, the muscle-specific overexpression [using the *24B-GAL4* driver (*6*)] of a single V-ATPase subunit (from a *UAS-Vha100-1* transgene) was sufficient to induce fully penetrant larval lethality approximately 2 days after hatching (Fig. 4C). Together, these results support the existence of a conserved functional interaction between miR-1 and the V-ATPase complex.

## DISCUSSION

The diverse range of cellular defects observed in muscles of animals lacking miR-1 had been so far attributed to numerous different targets (*12*), most of which are not conserved and many of which are likely secondary targets. Here, we identified a conserved set of direct targets that can explain the critical and seemingly pleiotropic role of miR-1 in muscle development and physiology. The mitochondrial fragmentation, protein aggregation, defects in cell-cell fusion, and conductance at the neuromuscular junction observed upon miR-1 loss can ultimately be related to defects in mitochondrial and lysosomal function. Dysfunction of these organelles affects trafficking and sorting of membrane proteins, autophagy, energy balance, and metabolic signaling through the TOR and AMPK pathways (*18*, *19*, *55*, *56*). These cellular processes can, in turn, be related to the regulon of functionally linked miR-1 targets comprising the V-ATPase complex and DCT-1/BNIP3, as shown here (fig. S7). Deregulation of the V-ATPase directly affects lysosomal function, whereas the primary effect of DCT-1/BNIP3 derepression is thought to be on mitochondria. However, given the tight connection between both organelles, defects in one have an impact on the other and result in a global loss of homeostasis of organelle dynamics and metabolic transfer (*57*).

Regarding TBC-7/TBC1D15 as a miR-1 target, a more significant contribution than the one that we observed here had been previously attributed (*22*). This is due to a stronger experimental overexpression in that study, relative to the up-regulation that we achieved by specific removal of miR-1 repression. However, *tbc-7* is significantly derepressed in the absence of miR-1 regulation. Moreover, given that it functions in shared processes with the V-ATPase, it is likely to contribute to the role of miR-1, although seemingly to a lesser extent than the other targets.

The miR-1 regulon comprises genes that are broadly, if not ubiquitously, expressed and that are essential for cell viability. This raises the question of why muscle cells need to specifically regulate these genes. We hypothesize that this relates to unique aspects of muscle morphology and physiology. Muscle cells have high energy demands and are thus particularly sensitive to regulators of mitochondrial homeostasis, as well as availability of metabolites that serve as energy sources (*58*). Moreover, high levels of respiration generate reactive oxygen species and increased mitochondrial damage, which makes the tight regulation of mitophagy a critical requirement (*59*). Muscle cells also display unique morphological characteristics: They are densely packed with myofibers and have a modified ER (sarcoplasmic reticulum) and plasma membrane to maximize conductance and contraction efficiency while maintaining stability under constant mechanical stress (*60*). In addition, despite not being a highly secretory tissue, muscle cells are particularly susceptible to ER stress (*61*) and may therefore require additional regulation of membrane and secreted proteins. miR-1–mediated control of ER, mitochondria, and lysosome biology may be necessary to achieve a delicate balance between different homeostatic processes in these highly specialized cells. The type of specific repression of ubiquitous genes mediated by miR-1 may provide an entry point to understand the origin of certain cellular vulnerabilities. For instance, germline mutations in the V-ATPase chaperone VMA21 cause a specific myopathy in humans (*62*) but spare most other cell types. On the basis of our findings, we hypothesize that this may be connected to the muscle-specific repression of V-ATPase subunits via miR-1.

miRNAs are repressors of gene expression, and hence, their biological functions stem from lowering the levels of their targets. Here, we show that miR-1 represses many subunits of a multiprotein complex, but this repression of individual components is required to promote correct function of the complex. Our work reveals a need for miR-1–mediated repression to ensure adequate levels, dynamics of production, and/or stoichiometry of the different subunits to achieve the correct assembly of the V-ATPase complex. Many V-ATPase subunits are transcriptionally coregulated by bHLH-Zip transcription factors of the MITF family, mostly acting downstream of the TOR pathway (*63*). In *C. elegans,* more than half of *vha* genes occur in operons with shared transcriptional regulation. Our work suggests that transcriptional regulation alone is not sufficient for establishing conditions for correct V-ATPase assembly in muscle cells, but this requires in addition the posttranscriptional regulation provided by miR-1. In addition to uncovering a muscle cell vulnerability, the results presented here point to a previously unknown role for miRNAs in coordinating assembly of multisubunit complexes.

## MATERIALS AND METHODS

### Strain maintenance

All *D. melanogaster* stocks were maintained under standard conditions at 18° or 25°C. All *C. elegans* strains were maintained on nematode growth media (NGM) plates seeded with OP50 bacteria at 20°C as previously described (*64*), unless indicated otherwise. A complete list of strains used in this study is presented in table S1.

### Generation of transgenic strains

Standard microinjection procedures were used to generate transgenic worms with extra chromosomal arrays (*65*). Briefly, DNA was injected as complex arrays in the gonads of N2 young adults. Injection mixes contained the plasmid of interest (1 to 5 ng/μl) and a similar amount of the coinjection marker *ttx-3p::mCherry*, in addition to sonicated genomic DNA (100 ng/μl) from *Escherichia coli* OP50. For VMA21 overexpression, WRM065bD07 fosmid (5 ng/μl) containing *R07E5.7/vma21* gene was injected. MLC1955 [*lucEx1114(WRM065bD07, ttx-3p::mCherry)*] and MLC1956 [*lucEx1115(WRM065bD07, ttx-3p::mCherry)*] lines were isolated. For *mir-1(luc108)* rescue, WRM0637aE07 fosmid (1 ng/μl) containing *mir-1* gene was injected in MLC2243 young adults. MLC2526 [*lucEx1353(WRM0637aE07; ttx-3p::mCherry); rmIs133 [unc-54p::Q40::YFP]; mir-1(luc108) I*], MLC2527 [*lucEx1354(WRM0637aE07; ttx-3p::mCherry); rmIs133 [unc-54p::Q40::YFP]; mir-1(luc108) I*], and MLC2528 [*lucEx1355(WRM0637aE07; ttx-3p::mCherry); rmIs133 [unc-54p::Q40::YFP]; mir-1(luc108) I*] lines were isolated.

Homology-directed genome editing using Cas9, CRISPR RNA (crRNA), and trans-activating crRNA (tracrRNA) ribonucleoprotein complexes in vitro assembled was performed as previously described (*66*). Briefly, purified Cas9 and synthetic RNAs were preincubated at 37°C for 15 min to enable complex formation and injected into the gonad of young adult worms. Alt-R CRISPR-Cas9 crRNA targeting the DNA sequences available in table S2 were obtained from Integrated DNA Technologies. *mir-1(luc108)* was created by deleting 117 base pairs (bp) of the *mir-1* locus, removing the whole-sequence coding for *mir-1* hairpin. *Vha-1(luc132)*, *vha-8(luc135)*, *vha-11(luc130)*, *vha-13(luc133)*, and *vha-14(luc134)* were created by replacing the miR-1 binding site (ACATTCCA) with the restriction site of the enzyme Not I (GCGGCCGC) in their 3′UTRs. *vha-12(luc139)* was created by replacing the three miR-1 binding sites with the restriction site of the enzymes Not I, Bam HI (GGATCC), and Eco RI (GAATTC) in its 3′UTR. *dct-1(luc145)* was created by replacing the two miR-1 binding sites with the restriction site of the enzyme Not I in its 3′UTR. *tbc-7(luc179)* was created by replacing the two miR-1 binding sites with the restriction sites of the enzymes Not I and Bam HI in its 3′UTR. *vha-1(luc161)* was created by deleting 454 bp of the *vha-1* locus, removing most of the coding sequence. As this deletion was lethal in homozygosis, this allele was balanced with the hT2 balancer.

### L1 synchronization

For the phenotypic characterization of *mir-1* mutant animals, 10 to 15 young adults were placed in unseeded 6-cm NGM plates with a bit of OP50 bacteria as food source. After 16 hours at 20°C, L1 larvae were collected and proceed to be imaged. For experiments with starved L1s, 15 to 20 young adults were bleached using hypochlorite solution (1% NaOCl and 1 M NaOH) in unseeded 6-cm NGM plates. Eggs were then washed three times with M9 buffer (22 mM KH_2_PO_4_, 42 mM Na_2_HPO_4_, 86 mM NaCl, and 1 mM MgSO_4_) and incubated at 20°C for 16 hours. Starved nutritional state is indicated in the corresponding figure legends.

### Plasmid construction

All constructs were generated by standard molecular cloning procedures with restriction digest, PCR, and Gibson assembly. The coding sequences in the constructs were verified by Sanger sequencing. Plasmid maps and sequences are available upon request.

### Transcriptome profiling

MLC2232 [*lucEx1207(myo-3p::YFP)*] and MLC2277 [*lucEx1207(myo-3p::YFP); mir-1(luc108)* I] worms were cultivated on five 10 cm peptone-enriched plates seeded with concentrated HB101 *E. coli* bacteria. Both strains were synchronized through two cycles of bleaching. On the first round, adult worms were bleached using hypochlorite solution, and isolated eggs were washed three times in M9 buffer and hatched for 14 hours in M9 buffer at 20°C, with gentle agitation. The next day, 75,000 starved L1s were plated on 150-mm peptone-enriched plates with concentrated HB101 and incubated at 21°C for ~52 hours. As the worms reached the stage of young adults, the population was closely monitored, and worms were collected when most of the population had eggs in their gonads. Collected worms were washed with ice-cold M9 buffer to remove residual bacteria and bleached with hypochlorite solution to release the eggs used for transcriptome profiling. Collected embryos were incubated at 25°C for ~300 min when ~70% of the population reached 1.5-fold stage and *myo-3p::YFP* expression was visible. The developmental stage of embryos was monitored using a Zeiss Axio Imager.Z2 with scientific complementary metal-oxide semiconductor (sCMOS) camera, a Sola SM2 solid-state white-light excitation system, and a 40×/1.3 EC plan-neofluar oil differential interference contrast (DIC) objective. The embryo suspension was concentrated to 0.5 ml by centrifugation at 1200 relative centrifugal force (rcf) for 1 min. To dissociate the eggshells, 0.5 ml of chitinase solution (2 mg/ml; Sigma-Aldrich; catalog no. C6137) was added to the embryo suspension and incubated for 30 min on ice with periodical shaking. A total of 100 μl of pronase solution (15 mg/ml; Sigma-Aldrich; catalog no. P6911) was then added to the sample and a 2.5-ml syringe fitted with a 21G needle was used to dissociate the embryos. The suspension was repeatedly passed through the needle until ~80% of embryos were dissociated. The digestion reaction was stopped by adding complete L-15 medium [L-15 no phenol red (Gibco; catalog no. 21083027); 10% fetal bovine serum, penicillin (50 U/ml), and streptomycin (50 μg/ml; Sigma-Aldrich; catalog no. P4458)], and cell suspension was filtered on a 5-μm cell strainer (pluriStrainer; catalog no. 43-10005-40) to remove undissociated embryos or cell aggregates. To label the dead cells, SYTOX AADvanced (Invitrogen; catalog no. S10349) was added to the sample to a final concentration of 1 μl/ml; the sample was incubated for 5 min and protected from light, and fluorescence-activated cell sorting (FACS) was performed on a Sony SH800, equipped with 488- and 561-nm lasers. Sorting was performed using 100-μm microfluidic chips (Sony, catalog no. LE-C3210) with standard settings and 5000 *myo-3p::YFP*–positive cells were collected in TRIzol (Invitrogen, catalog no. 15-596-018) for both MLC2232 and MLC2277 strains in duplicate.

To analyze gene expression profiles, mRNA libraries were prepared with the QuantSeq 3′ mRNA library prep kit FWD (Lexogen) and then sequenced with Illumina, including unique molecular identifiers (UMIs) and dual indexing. Specifically, RNA was extracted using the Direct-zol RNA Microprep Kit (Zymo Research, catalog no. R2060) reagent according to the manufacturer’s instructions. Yielding about 10 ng total RNA per sample, libraries were prepared following the QuantSeq instructions for low-input samples. After oligo-dT–primed reverse transcription, libraries were subjected to RT-qPCR in the presence of SYBR green until sufficient amplification was achieved, which required 21 cycles. Amplified libraries were pooled and sequenced on an Illumina NovaSeq SP PE150.

UMIs were first extracted with UMI tools (*67*). QuantSeq raw reads were then processed by trimming adaptors and first 4 bp of low quality and mapped to the *C. elegans* genome (WBcel235) using STAR aligner (*68*) with the parameter max_IntronL = 50000. Duplicated mapped reads were discarded with UMI tools, and UMI counts were quantified using HTSeq count (*69*). Differential expression between conditions was analyzed using the DESeq2 package (*70*). For analyzed and normalized mRNAseq data, see data file S1. The gene set enrichment analysis was done using R package “enrichplot.” Gene category enrichment analysis was performed using the web application WormCat (*71*) (http://wormcat.com).

### Reverse transcription followed by quantitative polymerase chain reaction

mRNA quantification of *mir-1*–predicted target genes in *C. elegans* was assessed via RT-qPCR following the procedure described in (*72*). Briefly, 10 starved or fed synchronized L1s were transferred to 2 μl of lysis buffer [5 mM tris-HCl (pH 8.0), 0.25 mM EDTA, proteinase K (1 mg/ml), 0.5% Triton X-100, and 0.5% Tween 20] using an eyelash. These samples were subjected to 10-min digestion at 65°C before heat inactivation of proteinase K for 1 min at 85°C. Genomic DNA was removed by incubating the samples with deoxyribonuclease I [DNase I; New England Biolabs (NEB), catalog no. M0303S] for 10 min at 37°C. Then, DNase I was inactivated by adding 25 mM EDTA and incubating the samples for 10 min at 65°C. Next, crude lysates were reverse-transcribed (Thermo Fisher Scientific, catalog no. 4368814) before performing qPCR using the GoTAQ qPCR Mastermix (Promega, catalog no. A6001) according to the manufacturer’s instructions. Relative expression was calculated according to the ΔΔCq method using *cdc-42* as a reference gene.

For mRNA quantification of *D. melanogaster Vha* subunits, 100 newly hatched L1s of the indicated genotype were transferred to 500 μl of TRIzol reagent (Thermo Fisher Scientific, catalog no. 15596026) and homogenized using an electric pestle tissue grinder for 2 to 3 min. Total RNA was isolated following the manufacturer’s instructions, and genomic DNA was removed by incubating with DNAse I (NEB, catalog no. M0303S). Samples (1 μg of total RNA) were then reverse-transcribed using the Superscript IV Reverse Transcriptase (Thermo Fisher Scientific, catalog no. 18090010), with oligo-dT as a primer. The resulting complementary DNA (cDNA) was used for qPCR, using the GoTAQ qPCR Mastermix (Promega, catalog no. A6001) according to the manufacturer’s instructions. For each sample, three technical replicates and an RT control were performed. The SD between technical replicates was always smaller than 0.5. In each reaction, a cDNA standard sample was included, and its Cq value was checked not to differ by more than 0.5 compared to the same standard sample used in the calibration curve of each gene. Relative expression was calculated according to the ΔΔCq method, using *Actin42a* and *Ef1*α*2* as reference genes. A complete list of primer sequences is provided in table S3 and raw Cq values in table S4.

### Cryo-preparation of *C. elegans*

N2 and MLC1384 [*mir-1(luc108)* I] animals were cultivated on three 15-cm peptone-enriched plates seeded with concentrated HB101 *E. coli* bacteria. Adult worms were bleached using hypochlorite solution, and isolated eggs were washed three times in M9 buffer, as described above. After approximately 16 hours, a pellet of L1 worms, containing 5% bovine serum albumin (fraction 5) in M9 buffer was transferred into the 100-μm cavity of a 3-mm aluminum specimen carrier. This carrier was sandwiched with a flat 3-mm aluminum carrier and immediately frozen under high pressure in an HPF Compact 01 (Engineering Office M. Wohlwend GmbH). The frozen samples were subsequently transferred into a Leica EM AFS-2 freeze substitution unit (Leica Microsystems). Over a period of 5 days, samples were substituted in a medium of acetone containing 2% osmium tetroxide (Electron Microscopy Sciences), 0.2% uranyl acetate (Merck), and 5% water. Freeze substitution was performed according to the following protocol: 60 hours at −90°C, warm-up at a rate of 2°C per hour to −54°C, 8 hours at −54°C, warm-up at a rate of 5°C per hour to −24°, 15 hours at −24°C, warm-up at a rate of 6°C per hour to 20°C, and 5 hours at 20°C. At 20°C samples were taken out and washed three times in anhydrous acetone. To enhance contrast, the samples were stained using 1% thiocarbohydrazid (Sigma-Aldrich) for 20 min at room temperature, 2% osmium tetroxide for 20 min at room temperature, and 2% uranyl acetate for 20 min at 60°C (three times 10-min washing steps after each staining step). Next, samples were infiltrated with Agar 100 Epoxy resin (Agar Scientific), in a graded series of acetone and resin over a period of 2 to 3 days. Polymerization took place at 60°C.

Ultrathin sections were cut using a Leica UCT ultramicrotome (Leica Microsystems) at a nominal thickness of 70 nm and picked up on 100-mesh Cu/Pd grids (Agar Scientific) previously coated with a supporting film of formvar (Agar Scientific). Examination regions on the sections were selected at random, inspected with an FEI Morgagni 268D (FEI, the Netherlands) operated at 80 kV. Digital images were acquired using a 11-megapixel Morada charge-coupled device (CCD) camera (Olympus-SIS, Germany).

### Immunostaining

To analyze the sarcomere organization of *mir-1* mutant animals, N2 and MLC1384 [*mir-1(luc108)* I] were cultivated on four 9-cm NGM plates seeded with OP50 *E. coli* bacteria at 20°C. Worms from different stages were washed three times with M9 and then fixed in RFB solution (80 mM KCl, 20 mM NaCl, 10 mM EGTA, and 5 mM spermidine) supplemented with 2% of formaldehyde. Worms were freeze-thawed three times, transferring from liquid nitrogen to 37°C and incubated for 30 min at room temperature on a nutator. Worms were then washed in TTE buffer [100 mM tris (pH 7.5), 1% Triton X-100, and 1 mM EDTA (pH 8)] and incubated in TTE buffer supplemented with 1% β-mercaptoethanol for 4 hours at 37°C on the nutator. Then, samples were washed in BO_3_ buffer (10 mM H_3_BO_3_, 10 mM NaOH, and 2% Triton X-100) and incubated in BO_3_ buffer supplemented with 10 mM dithiothreitol for 15 min at 37°C on the nutator. Next, they were washed again and incubated in BO_3_ buffer supplemented with 0.3% H_2_O_2_ for 15 min at room temperature. After washing one last time with BO_3_ buffer, samples were incubated with blocking buffer [1× phosphate-buffered saline (PBS), 0.25% Triton X-100, 40 mM NaN_3_, and 0.2% gelatin] for 30 min at room temperature. To visualize the heavy chain A of myosin, mouse 5-6 antibody [Epstein, Developmental Studies Hybridoma Bank (DSHB)] was diluted 1:200 in PGT buffer (1× PBS, 0.25% Triton X-100, 40 mM NaN_3_, and 0.1% gelatin), whereas to visualize the M-lines, we used rabbit UNC-89 antibody (1:200 in PGT buffer, a gift from G. Benian). Samples were incubated overnight at 4°C on the nutator, and then they were washed five times with washing buffer (1× PBS and 0.25% Triton X-100) for 30 min at room temperature. Anti-mouse immunoglobulin G (IgG) conjugated with Alexa Fluor 488 (1:200, Abcam, catalog no. ab150113) or anti-rabbit IgG conjugated with Alexa Fluor 647 (1:200, Abcam, catalog no. ab150083) was added to the samples, and they were incubated in the dark overnight at 4°C on the nutator. Last, samples were washed five times with washing buffer and ProLong Gold Antifade reagent with 4′,6-diamidino-2-phenylindole (DAPI) was added to worm pellets.

### Behavior and morphological assays

#### Mitochondria network

For analysis of mitochondrial network organization in *C. elegans* body-wall muscles, the strain SJ4103 [*zcIs14(myo-3::GFP(mit))*] was used (*73*). Images from synchronized L1s were deconvoluted with classic maximum likelihood estimation (CMLE) algorithm, 40 iterations, and 15 signal/noise ratio (Huygens deconvolution software, Scientific Volume Imaging) and quantified using a two-step approach: We applied a three-dimensional (3D) Laplacian of Gaussian filter and thresholded the resulting image (Fiji-ImageJ software, version 1.52p). This binary image was used to create surface objects in Imaris software (version 9.3.1, Bitplane), allowing for measurement of their area, volume, and sphericity and export the values for statistical analysis. An average of 150 to 200 mitochondria objects (*n*) was detected per animal (*N*). Median values for area, volume, and sphericity were extracted per animal. Images with less than 50 objects detected were excluded for further analysis.

#### ATP levels

For measuring ATP levels in the body-wall muscle of *C. elegans*, the strain GA2001 [*wuIs305 (myo-3p::Queen-2m)*] expressing the ratiometric Queen-2m ATP biosensor was used as previously described (*74*). Images from fed and synchronized L1s were further processed in Fiji-ImageJ software. After subtracting the background, an average intensity projection of the whole Z-stack was applied for both 405- and 488-nm excitation channels. Then, the ratio 405 excitation/488 excitation was calculated by dividing the 32-bit resulting images. Three independent regions of interests were selected, and the median of them was used for plotting and statistical analysis.

#### Protein aggregation

To assess proteostasis defects, synchronized and fed L4s animals carrying the rmIs133 [*unc-54p::Q40::YFP*] transgene in their body-wall muscles were imaged (*75*). The resulting images were further processed with Fiji-ImageJ software: A maximum-intensity Z-stack projection was extracted, followed by thresholding. Particles with a circularity between 0.5 and 1.0 and size bigger than four pixels were counted as aggregates.

#### pm3D cell-cell fusion

pm3DL and R fusion was assessed by the expression of a pm3DR-specific membrane marker (*mir-4813p::myr::GFP*). Images from synchronized and fed L1s were deconvoluted with CMLE algorithm, 40 iterations, and 15 signal/noise ratio (Huygens deconvolution software, Scientific Volume Imaging). To obtain representative cross sections of pm3D, we used an ImageJ-Macro, which allowed us to automatically export three equidistant cross sections perpendicular to a manually drawn line along the respective region. For the export, the “Reslice” function was used. Cross sections were analyzed blindly. Macro is available upon request.

#### Electropharyngeograms

Electropharyngeograms (EPGs) were used as a readout of pharyngeal pumping in N2, MT17810 [*mir-1(n4102)* I], and MLC1384 [*mir-1(luc108)* I]. Approximately 22 hours before recording, about 40 L4s were transferred onto an NGM plate seeded with OP50 at 20°C. Before starting the recordings, the worms were washed with 1.2 ml of M9 to remove pieces of agar and food. After removing 1 ml of the supernatant, 1 ml of 10 mM serotonin (diluted in M9) was added to the worms. Worms were incubated in serotonin for 40 min before loading them on the ScreenChip System (SC40, InVivo Biosystems, catalog no. SKC101). For acquiring EPG data and data analysis, we used the software supplied by InVivo Biosystems (NemAquire and NemAnalysis). Pharyngeal pumping of each worm was recorded for 2 min. All recordings with a signal/noise ratio level below 3.0 were discarded. For comparing the peak-triggered average between different worm strains, we used MATLAB in collaboration with J. Riedl [Zimmer laboratory, Research Institute of Molecular Pathology (IMP)].

#### Lysosomal marker and pH sensor

For assessing lysosomal pH and morphology in the body-wall muscle of *C. elegans*, we cloned a LAMP1 fusion to a ratiometric pH sensor (RpH-LAMP1) (*48*). This consists of the tandem fusion of the superecliptic pHluorin to mCherry and to the N-terminal part of the lysosomal-specific protein LAMP1, from mouse. This was placed under control of the *myo-3* promoter. We generated the strain MLC2465 [*oxIs322 II; unc-119(ed3) III; lucEx1311(myo-3p::RpH-LAMP1-3xFLAG::unc-54 3′UTR; ttx-3p::mCherry)*] expressing this reporter and crossed it to *mir-1(luc108)* animals. Fed and synchronized L1s were mounted on a 5% bacto-agar pad and immobilized in 50 mM NaN_3_. Images were acquired using an Olympus Inverted Spinning Disc Confocal (Olympus IX83 series, Tokyo, Japan), Hamamatsu Orca Fusion CMOS camera, 100×/1.45 Oil UPLXApo WD objective, and 100% power and 500-ms exposure time of lasers 488 and 561 nm. Z-stack space 0.1 μm was used. Images were further processed in Aivia software (https://aivia-software.com) to extract fluorescence ratio (indicative of pH) and volume of individual lysosomes in 3D. Background subtraction and size threshold were performed to segment the different vesicles, and then a watershed was applied to separate them. Mean intensity parameters from both pHluorin and mCherry channels were exported and used to calculate the pHluorin/mCherry ratio. An average of 30 to 50 lysosomes (*n*) was detected per animal (*N*). Median values for pHluorin/mCherry ratio and volume per animal were plotted and considered for statistical analysis.

#### Drosophila larval survival assay

Adult flies were allowed to lay embryos on apple juice agar plates for 4 hours at 25°C. On the next day, 10 newly hatched L1s of the desired genotype were collected and placed on fresh apple juice agar plates with food (paste of baker’s yeast and water). Every 24 hours, alive larvae were transferred to fresh plates with food, while dead larvae were removed and scored. The experiment was followed until all the larva either turned into a pupa or died.

For the overexpression of the *Vha* subunit, we crossed an available *UAS-Vha100-1* strain (BL-39669) (*76*) with the pan-mesodermal *how24B-Gal4* driver strain (BL-1767) (*6*). The BL-39669 strain contains also a recessive lethal loss of function allele of *Vha100-1* (*Vha100-1^1^*); therefore, half of the progeny from the cross was expected to be heterozygous for this mutation. Because we did not know what effect this could have on the survival of larvae, we genotyped every animal we assayed for presence of this allele. We saw that, independently of the presence of the *Vha100-1^1^* allele, all larvae died at approximately day 2 of the assay.

#### Drosophila larval length

Adult flies were allowed to lay embryos on apple juice agar plates for 4 hours at 25°C. On the next day, 10 newly hatched L1s of the desired genotype were collected and placed on fresh apple juice agar plates with food (paste of baker’s yeast and water). After 24 hours, the larvae were collected and anesthetized in a small etherization cage using a 1.5-ml microcentrifuge tube, as previously described (*77*). Larvae were then mounted in a slide with a 2.5% bacto-agar pad and imaged in a wide-field upright microscope (Zeiss Axio Imager.Z2 with sCMOS camera, 5× objective). Length of the larvae was measured using Fiji-ImageJ software.

### Spinning disk confocal microscopy

L1 or L4 worms were mounted on a 5% bacto-agar pad and immobilized in 50 mM NaN_3_. Images were acquired using a Visiscope Spinning Disc Confocal (Visitron Systems GmbH, Puching, Germany) with PCO Edge 4.2-m sCMOS camera, CFI plan Apo lambda 100×/1.45 oil (or 10×/0.45 air for protein aggregation), and 100% power of lasers 405 nm and 120 mW, 488 nm and 200 mW, and/or 561 nm and 150 mW. A Z-stack space of 0.1 μm was used, except for the analysis of protein aggregation that a Z-stack space of 1 μm was used and for the measurement of lysosomal pH where a different setup was used (see the “Lysosomal marker and pH sensor” section).

### Data analysis

Statistical tests were performed in Prism v7 or 8 (GraphPad Software Inc.) and are described for each figure. In general, to identify outliers, the robust regression and outlier removal (ROUT) test was applied. To assess whether the data were following a normal distribution, three independent normality tests (D’Agostino and Pearson, Shapiro-Wilk, and Kolmogorov-Smirnov) were performed. If the distribution was normal, then one-way analysis of variance (ANOVA) was performed for comparisons across multiple independent samples, using Tukey’s multiple comparisons correction. If not, then Kruskal-Wallis test using Dunn’s multiple comparisons correction was used. Median with interquartile range was plotted for all behavior analysis. For RT-qPCR analysis, mean with standard SEM was plotted and an unpaired *t* test was performed.

## Notes

View/request a protocol for this paper from *Bio-protocol*.
