# Supplementary material for: miR-1 sustains muscle physiology by controlling V-ATPase complex assembly
Source: Sci Adv. 2021 Oct 15;7(42):eabh1434. doi: 10.1126/sciadv.abh1434 (PMC8519577; doi:10.1126/sciadv.abh1434)
Supplement: Supplementary file 1 — Figs. S1 to S7 Tables S1 to S4 [file sciadv.abh1434_sm.pdf]

Science Advances
AAAS

Supplementary Materials for

miR-1 sustains muscle physiology by controlling V-ATPase complex assembly

Paula Gutiérrez-Pérez, Emilio M. Santillán, Thomas Lendl, Jingkui Wang, Anna Schrempf, Thomas L. Steinacker, Mila Asparuhova, Marlene Brandstetter, David Haselbach, Luisa Cochella*

*Corresponding author. Email: mcochel1@jhmi.edu

Published 15 October 2021, *Sci. Adv.* **7**, eabh1434 (2021)
DOI: 10.1126/sciadv.abh1434

The PDF file includes:

Figs. S1 to S7
Tables S1 to S4

Other Supplementary Material for this manuscript includes the following:

Data file S1

Supplementary Figures

A

	C. elegans	*D. melanogaster*	*D. rerio*	*H. sapiens*	
**V-ATPase subunit**					
**A**	***vha-13***	***vha68-2***	***atp6v1ab***	***atp6v1a***	
**B**	***vha-12***	***vha55***	***atp6v1ba***	***atp6v1b2***	
**C**	***vha-11***	***vha44***	***atp6v1c1a/b***	***atp6v1c1***	
**D**	***vha-14***	*vha36*	***atp6v1d***	*atp6v1d*	V_1
**E**	***vha-8***	***vha26***	*atp6v1e1*	*atp6v1e1/2*	
**F**	***vha-9***	*vha14*	*atp6v1f*	*atp6v1f*	
**G**	***vha-10***	*vha13*	*atp6v1g*	*atp6v1g1/2/3*	
**H**	***vha-15***	***vhaSFD***	***atp6v1h***	*atp6v1h*	
**a**	***vha-5***	***vha100-3***	***atp6v0a2***	***atp6v0a1***	
**c**	***vha-1/-2/-3***	*vha16*	***atp6v0ca***	***atp6v0c***	
**c”**	***vha-4***	*vhaPPA1*	***atp6v0b***	*atp6v0b*	V_0
**d**	***vha-16***	***vhaAC39-1***	***atp6v0d1***	*atp6v0d1/2*	
**e**	***vha-17***	*vhaM9.7*	*atp6v0e1*	*atp6v0e1/2*	
**Ac45**	***vha-19***	*vhaAC45*	*atp6ap1*	*atp6ap1*	
**PRR/M8.9**	-	*vhaM8.9*	***atp6ap2***	*atp6ap2**	
**Mitophagy (DCT-1/BNIP3)**					
	dct-1	*cg5059/bnip3*	***bnip3/bnip3l****	*bnip3l**	
**Autophagy (TBC-7/TBC1D15)**					
	tbc-7	***sky***	***tbc1d24***	***tbc1d15***	

B

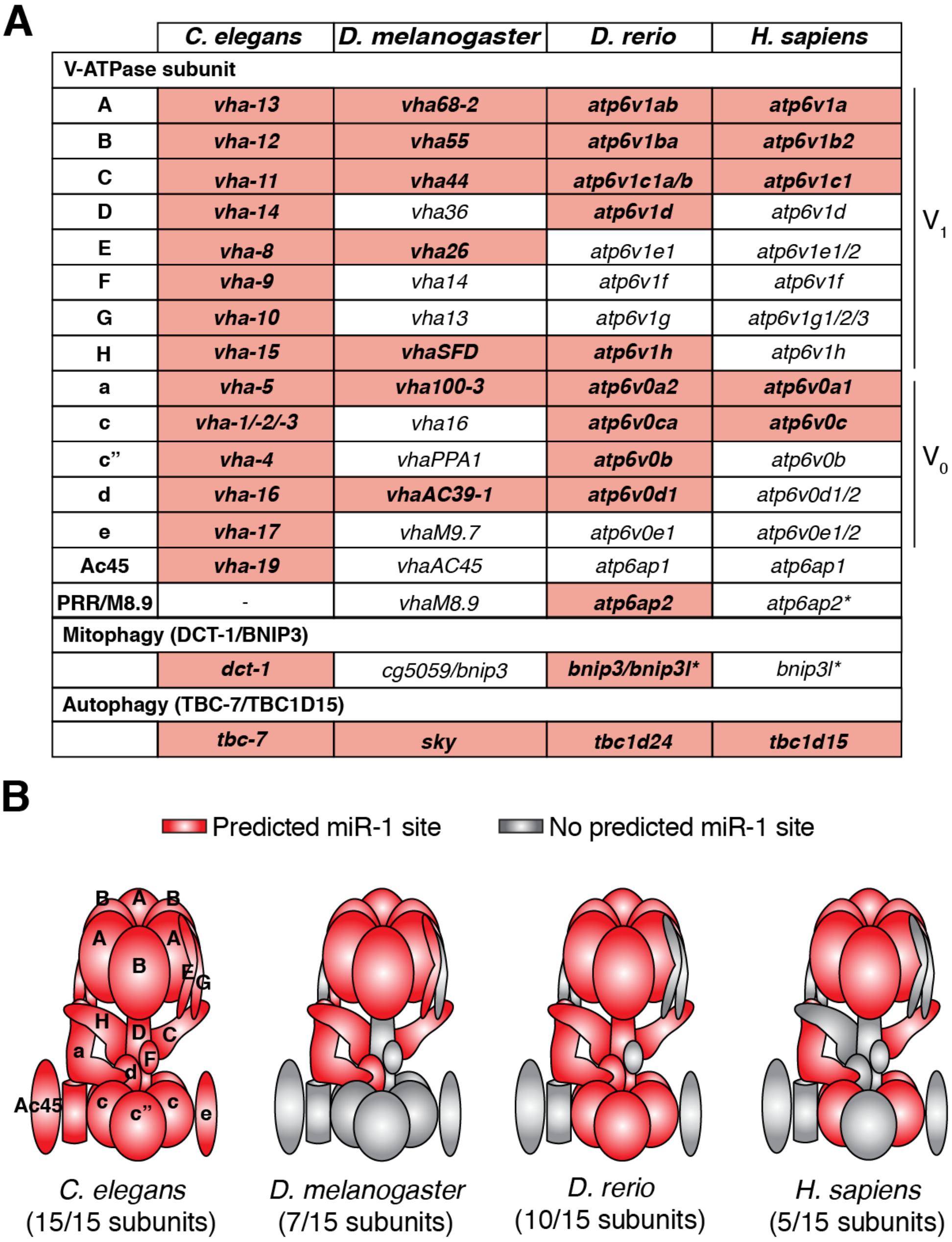

Figure S1. V-ATPase, DCT-1/BNIP3 and TBC-7/TBC1D15 are predicted miR-1 targets across animals. **(A)** Several subunits of the V-ATPase complex, *dct-1/bnip3* and *tbc-7/tbc1d15* have at least one miR-1 predicted binding site in their 3′ UTR (bold, red shade) in *C. elegans*, *D. melanogaster*, *D. rerio* and *H. sapiens* (*13*, *14*, *15*). Asterisks (*) refer to the presence of a predicted miR-133 binding site in *bnip3l* instead; in vertebrates, miR-1 and miR-133 are clustered together and co-expressed specifically in muscle. **(B)** Location of V-ATPase subunits that are predicted miR-1 targets; miR-1 regulation could affect subunits across the whole complex.

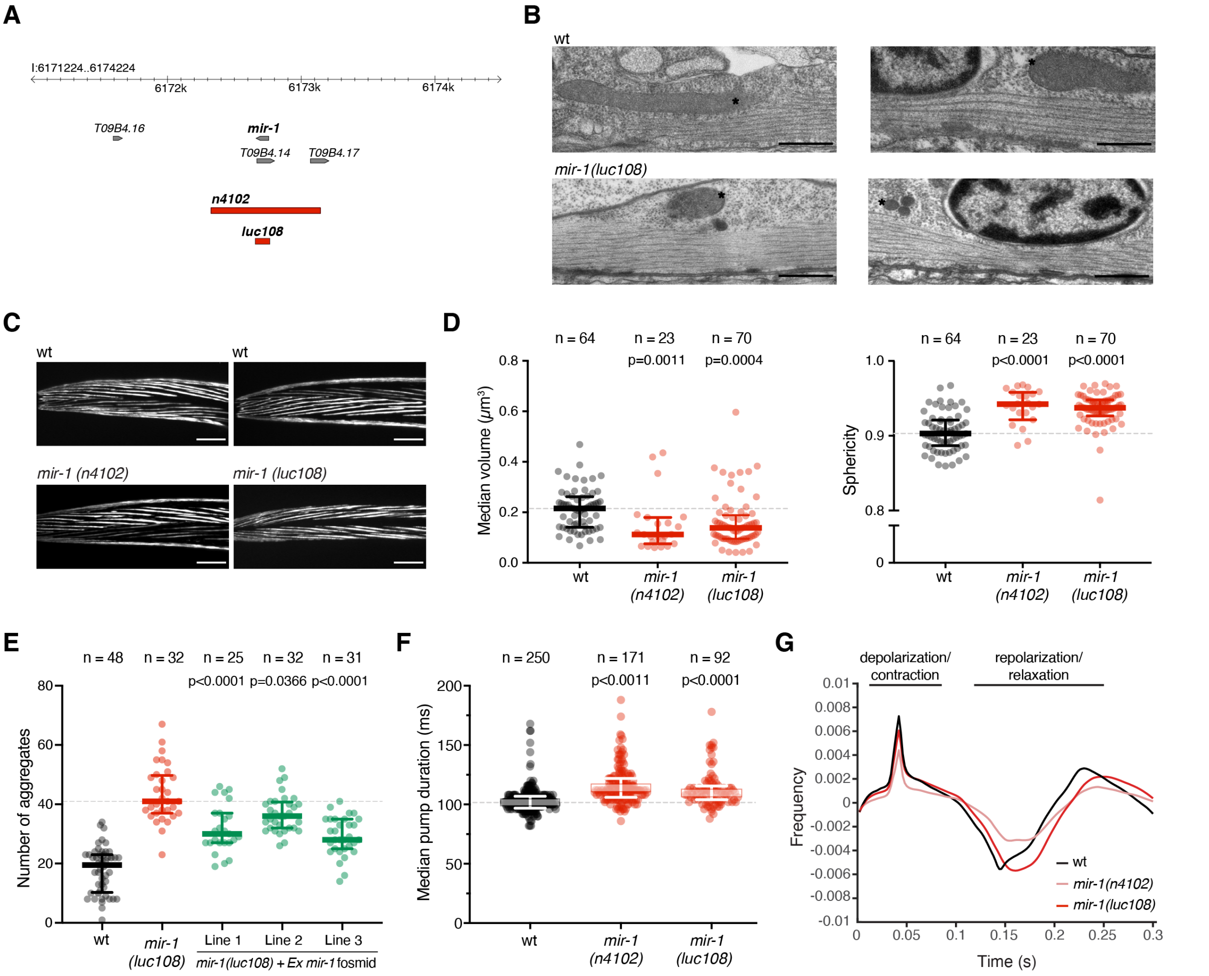

Figure S2. Absence of miR-1 causes diverse muscle defects in *C. elegans*. (A) A schematic depicting the *mir-1* locus and the two deletion alleles used in this study: *n4102* was obtained by EMS mutagenesis, *luc108* was generated using CRISPR/Cas9 for this study. **(B)** Representative electron microscopy images for both wt and *mir-1(luc108)* L1 larvae. The sarcomere structure is preserved. Asterisks (*) indicate mitochondria. Scale bars = 0.5 µm. **(C)** Representative fluorescence images of animals expressing MYO-3::GFP (maximum intensity projections of stacks through the whole animal). The typical parallel organization is preserved in both *mir-1(n4102)* and *mir-1(luc108)* L1s. Scale bars = 10 µm. **(D)** (left) Median mitochondria volume, extracted from confocal microscopy images of L1 larvae expressing mitochondrial GFP (see Fig. 1A and Methods). (right) Mitochondria sphericity was extracted from the same images. Mitochondria are smaller and more spherical in *mir-1* mutant animals, indicating fragmentation of the network. P-values by Kruskal-Wallis test are indicated. **(E)** Restoring *mir-1* levels in *mir-1(luc108)* animals significantly reduces the number of aberrant aggregates, in three independent lines. P-values (one-way ANOVA) are shown. Note: obtaining rescue lines for miR-1 has been overall challenging as it is easy to cause miR-1 overexpression, and this is extremely toxic. The balance between expressing enough miR-1 but not too much is difficult to achieve with transgenes. **(F)** Median duration of each pharyngeal pumping cycle was extracted from the EPGs analyzed in Fig. 1E using NemAcquire and NemAnalysis softwares. In *mir-1(n4102)* and *mir-1(luc108)* animals each pump is longer, causing the lower overall frequency (Fig. 1E). P-values by Kruskal-Wallis test are indicated. **(G)** Peak-triggered average of all pharyngeal pumping events for each genotype revealed a normal duration of the contraction phase, but a longer relaxation/recovery phase in *mir-1* mutant young adults, suggesting a reduced capacity of recovering after each contraction. n refers to number of animals analyzed.

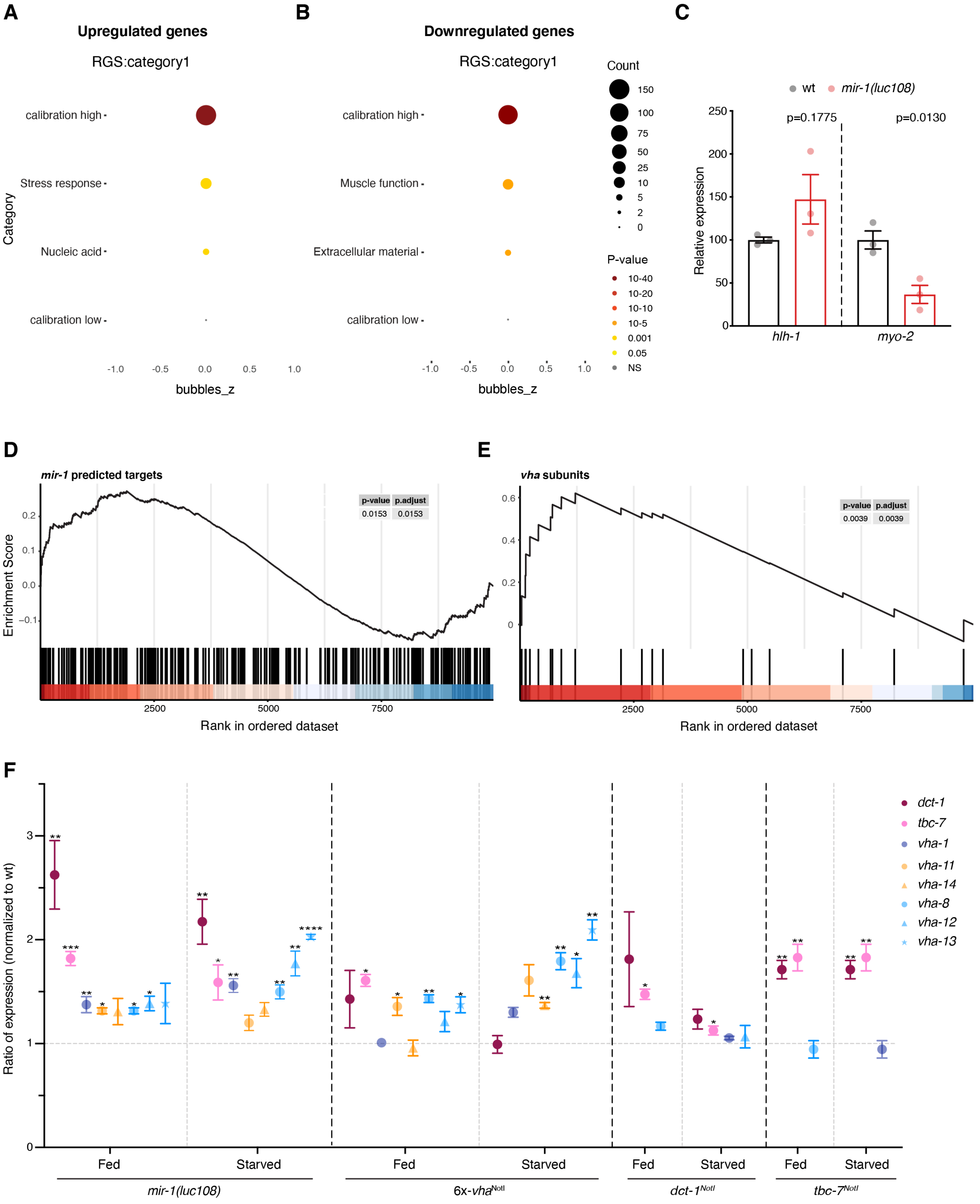

Figure S3. Multiple subunits of the V-ATPase complex are repressed by miR-1 in *C. elegans*. (A) and **(B)** Bubble charts generated by WormCat gene category enrichment analysis (see Methods) of upregulated (A) and downregulated genes (B) in *mir-1(luc108)* muscle cells. Transcripts that were upregulated (A) or downregulated (B) by at least 30% were used as input for this analysis (280 and 115 respectively). Bubbles report the number of genes corresponding to each of the enriched categories (size) and the associated p-values (color). **(C)** *mir-1(luc108)* animals show defects in muscle differentiation, as indicated by increased levels of the body wall muscle-master regulator (*hlh-1*) and decreased levels of pharyngeal myosin (*myo-2*). Three independent biological replicates were analyzed. Each biological replicate consists of 10 L1 starved animals. P-values by unpaired t-test are

shown**.** Error bars correspond to SEM. **(D)** and **(E)** Gene Set Enrichment Analysis (GSEA) profiles of *mir-1* predicted targets (D) and *vha* genes (E) in RNAseq data from sorted muscle cells of *mir-1(luc108)* vs. wt 1.5-fold embryos. There is a significant enrichment of *vha* genes in *mir-1* mutant animals. Vertical black lines indicate the position of each of the transcripts in the ranked, non-redundant differentially-expressed gene data set (red refers to upregulated, blue to downregulated). The black curve indicates the enrichment score, i.e. the running sum of the weighted enrichment score in GSEA. **(F)** Quantitative RT-qPCR on whole fed or starved L1 larvae of the indicated genotypes for *dct-1*, *tbc-7*, *vha-1, -8, -11, -12, -13* and *-14*. The mean ratio of three independent biological replicates normalized to the corresponding wt sample is plotted. Each biological replicate consists of 10 L1 animals. Each biological replicate was analyzed in technical triplicates (all qPCR data can be found in Table S4). Error bars correspond to SEM. P-values by unpaired t-test are shown (*<0.05, **<0.01, ***<0.001, ****<0.0001).
Note: The fact that RT-qPCRs are conducted on whole animals (and not isolated muscle) limits the sensitivity of the assay. Also, some of the transcripts we tried to detect were present at very low levels (in particular *dct-1*, see Table S4) and the magnitude of the changes are also modest, so in some cases clear trends do not reach significance. Signals obtained from starved animals were more reproducible than from fed animals (either due to better stage synchronization or due to food-related regulation).

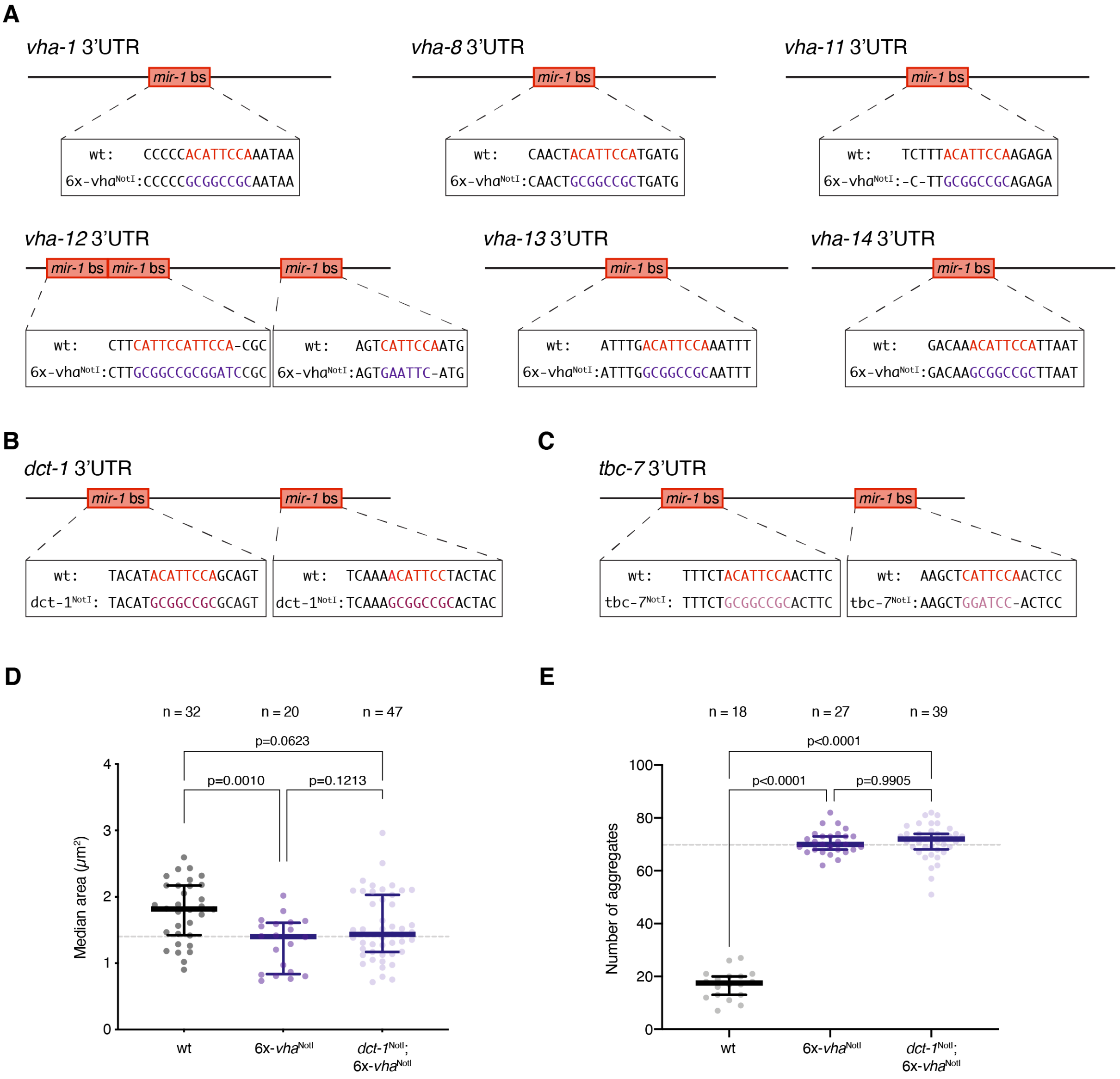

Figure S4. V-ATPase subunits and DCT-1 are functionally relevant targets of miR-1. (A) Schematic representation of the 3′ UTR sequence of wt and *6x-vha*^{NotI} animals. The *mir-1* binding sites (ACATTCC, red) in the 3′ UTRs of *vha-1, -8, -11, -12, -13 and -14* were replaced by NotI restriction sites (GCGGCCGC, purple), except for *vha-12* where the second and third *mir-1* binding sites were replaced by BamHI (GGATCC) and EcoRI (GAATTC) restriction sites, respectively. **(B)** Schematic representing the 3′ UTR sequence of wt and *dct-1*^{NotI} animals. The *mir-1* binding sites were replaced by NotI restriction sites. **(C)** Schematic representing the 3′ UTR sequence of wt and *tbc-7*^{NotI} animals. The *mir-1* binding sites were replaced by NotI and BamHI restriction sites. **(D and E)** *dct-1* and V-ATPase subunits are non-redundant targets of *mir-1*. **(D)** Median mitochondria area is similarly reduced in both *6x-vhaNotI* and *dct-1*^{NotI}; *6x-vhaNotI* animals. P-values by one-way ANOVA are indicated. wt data set is replotted from Fig. 2C (right). **(E)** *6x-vhaNotI* and *dct-1*^{NotI}; *6x-vhaNotI* L4 animals show a similarly impaired proteostasis, P-values (one-way ANOVA) are shown. n refers to number of animals analyzed.

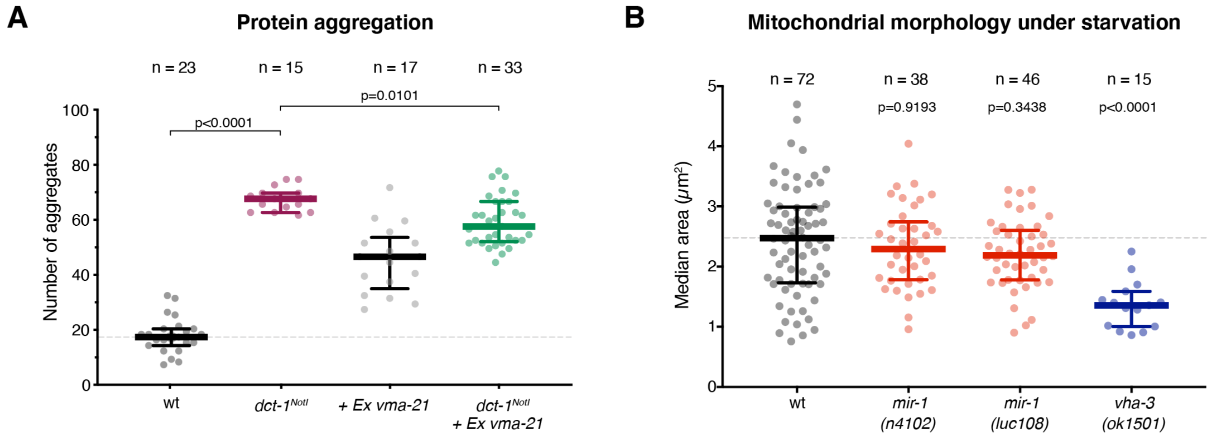

Figure S5. Loss of miR-1 affects the assembly of the V-ATPase complex. (A) Overexpression of VMA-21 does not rescue *dct-1*^{NotI} proteostasis defects. P-values by one-way ANOVA are shown. **(B)** Starvation rescues mitochondrial network fragmentation in *mir-1(n4102* and *luc108)* animals but not in *vha-3(ok1501)* mutants. P-values by one-way ANOVA are shown. n refers to number of animals analyzed.

A

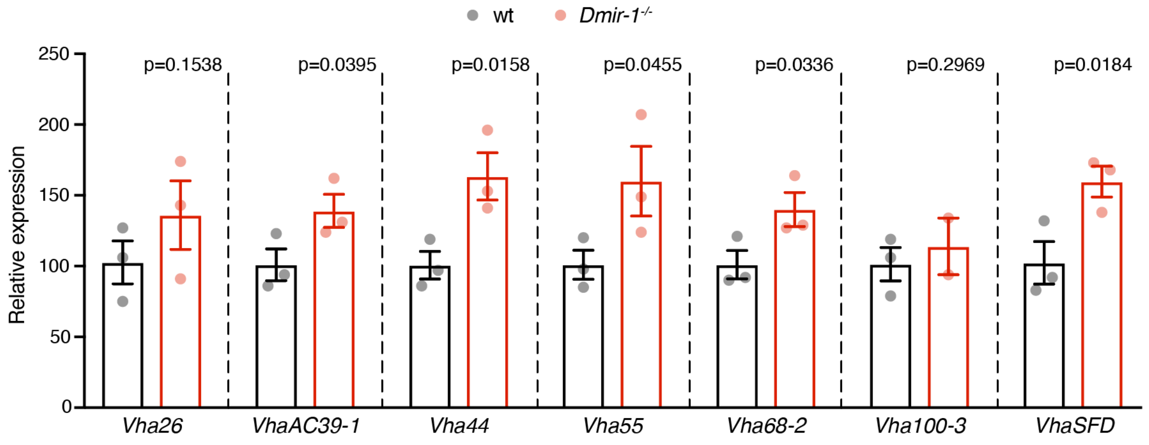

B

24B-GAL4, UAS-GFP::lacZ.nls

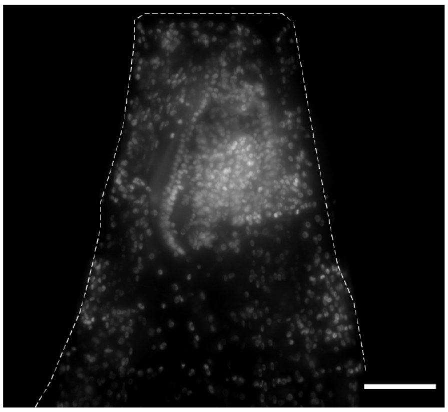

Figure S6. Multiple subunits of the V-ATPase complex are repressed by miR-1 in *Drosophila*. (A) Quantitative RT-qPCR for various *Vha* transcripts from whole *Drosophila* first instar larvae of the indicated genotypes; *Vha* transcripts are upregulated in the absence of miR-1. Three independent biological replicates, consisting of 10 larvae, were analyzed. P-values by unpaired t-test are shown. Error bars correspond to SEM. **(B)** Representative image of a 24-hour post-hatching *Drosophila* larva expressing GFP in muscle nuclei, driven by the same 24B-GAL4 driver used for *Vha100-1* overexpression in Figure 4C. Scale bar = 50µm.

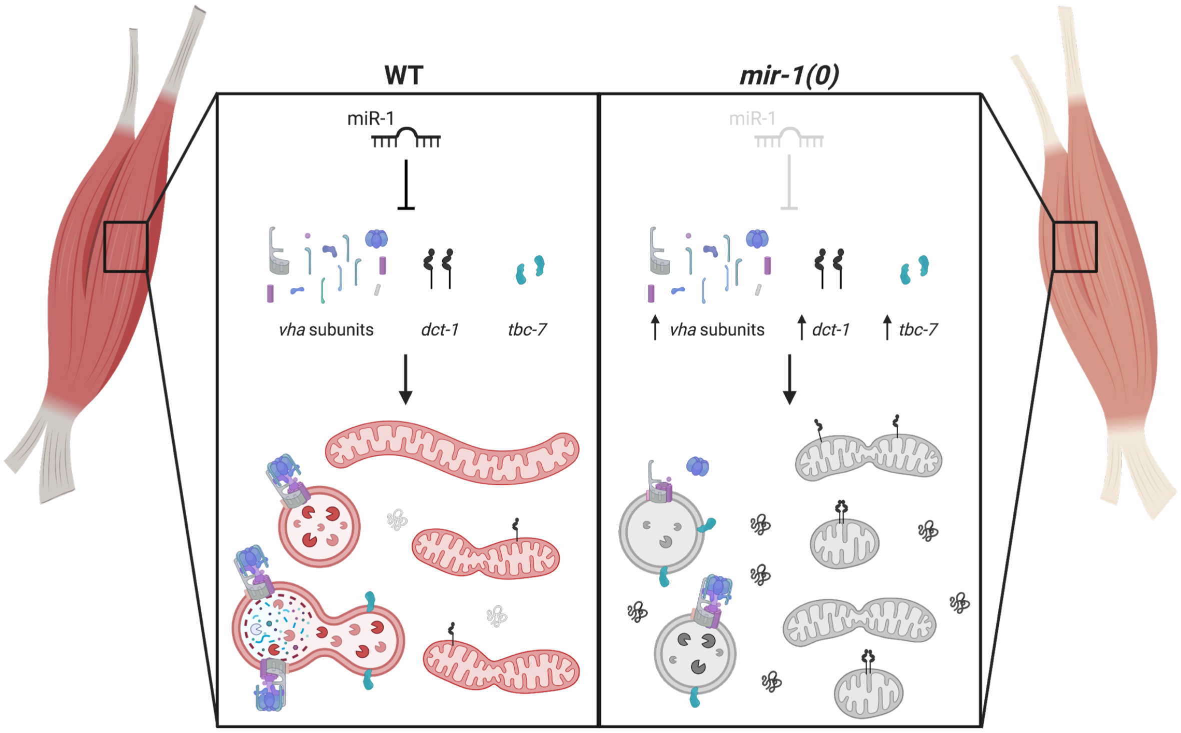

Figure S7. Model of miR-1 function in muscle cells*.* (left) Correct muscle physiology is achieved by miR-1 mediated repression of *vha* subunits, *dct-1* and *tbc-7* at the post-transcriptional level. This regulation enables the proper assembly of the V-ATPase complex and the levels of both DCT-1 and TBC-7, leading to balanced mitochondria and lysosome function. **(right)** In the absence of miR-1, the *vha* subunits, *dct-1* and *tbc-7* transcripts are upregulated leading to loss of function of the V-ATPase complex and to defects in the mitochondria-lysosome axis. This deregulation affects the mitochondria network, the production of energy, protein homeostasis and autophagy, which ultimately cause severe damage to muscle cells.

Supplementary Spreadsheet

Results of mRNA 3’ sequencing (Quant-seq) from muscle cells isolated from wild-type or *mir-1*-deficient embryos. Biological duplicates for each genotype are shown. Read numbers were normalized and genotypes were compared using DEseq2 (see methods). Log_2(fold change mir-1/wt) and associated p-values are reported for every detected gene.

Supplementary Tables

Table S1. Experiment Models: Organisms/Strains used in this study.

Experimental Models: Organisms/Strains		
Caenorhabditis elegans: Wild type Bristol isolate	Caenorhabditis Genetics Center	WB Strain: N2
Caenorhabditis elegans: mir-1(n4102) I. Outcrossed 6x	Caenorhabditis Genetics Center	MT17810
Caenorhabditis elegans: mir-1(luc108) I	This Study	MLC1384
Caenorhabditis elegans: lucEx421[mir-4813 1kb upstream p::myr::gfp::1kb unc-54 3'UTR downstream; ttx-3p::mCherry]	This Study	MLC603
Caenorhabditis elegans: lucEx421 [mir-4813 1kb upstream p::myr::gfp::1kb unc-54 3'UTR downstream; ttx-3p::mCherry]; mir-1(luc108) I	This Study	MLC1532
Caenorhabditis elegans: lucEx421 [mir-4813 1kb upstream p::myr::gfp::1kb unc-54 3'UTR downstream; ttx-3p::mCherry]; mir-1(n4102) I	This Study	MLC1614
Caenorhabditis elegans: aff-1(tm2214) II	Caenorhabditis Genetics Center	BP600
Caenorhabditis elegans: eff-1(hy21) II	Caenorhabditis Genetics Center	BP75
Caenorhabditis elegans: lucEx421 [mir-4813 1kb upstream p::myr::gfp::1kb unc-54 3'UTR downstream; ttx-3p::mCherry]; aff-1(tm2214) II	This Study	MLC1757
Caenorhabditis elegans: lucEx421 [mir-4813 1kb upstream p::myr::gfp::1kb unc-54 3'UTR downstream; ttx-3p::mCherry]; eff-1(hy21) II	This Study	MLC1685
Caenorhabditis elegans: zcIs14 [myo-3p::GFP(mit)]	Caenorhabditis Genetics Center	SJ4103
Caenorhabditis elegans: zcIs14 [myo-3p::GFP(mit)]; mir-1(n4102) I	This Study	MLC1652
Caenorhabditis elegans: zcIs14 [myo-3p::GFP(mit)]; mir-1(luc108) I	This Study	MLC1653
Caenorhabditis elegans: rmIs133 [unc-54p::Q40::YFP] X	Caenorhabditis Genetics Center	AM141
Caenorhabditis elegans: rmIs133 [unc-54p::Q40::YFP] X; mir-1(n4102) I	This Study	MLC2244
Caenorhabditis elegans: rmIs133 [unc-54p::Q40::YFP] X; mir-1(luc108) I	This Study	MLC1688/MLC2243
Caenorhabditis elegans: kagIs4[gfp::myo-3, V:12226816]	Kathrin Gieseler, France	KAG420
Caenorhabditis elegans: kagIs4[gfp::myo-3, V:12226816]; mir-1(n4102) I	This Study	MLC1772
Caenorhabditis elegans: kagIs4[gfp::myo-3, V:12226816]; mir-1(luc108) I	This Study	MLC1773
Caenorhabditis elegans: wuIs305 [myo-3p::Queen-2m]	Caenorhabditis Genetics Center	GA2001
Caenorhabditis elegans: wuIs305 [myo-3p::Queen-2m]; mir-1(n4102) I	This Study	MLC1902
Caenorhabditis elegans: wuIs305 [myo-3p::Queen-2m]; mir-1(luc108) I	This Study	MLC1903
Caenorhabditis elegans: luc130(vha-11::3'UTRmir-1bs->NotI) IV	This Study	MLC1774
Caenorhabditis elegans: luc132(vha-1::3'UTRmir-1bs->NotI) III	This Study	MLC1777
Caenorhabditis elegans: luc133(vha-13::3'UTRmir-1bs->NotI) V	This Study	MLC1778
Caenorhabditis elegans: luc134(vha-14::3'UTRmir-1bs->NotI) III	This Study	MLC1779

Caenorhabditis elegans: luc132(vha-1::3'UTRmir-1bs->NotI) III; luc130 (vha-11::3'UTRmir-1bs->NotI) IV	This Study	MLC1786
Caenorhabditis elegans: luc135(vha-8::3'UTRmir-1bs->NotI) IV	This Study	MLC1801
Caenorhabditis elegans: luc132(vha-1::3'UTRmir-1bs->NotI) III; luc130 (vha-11::3'UTRmir-1bs->NotI) IV; luc133(vha-13::3'UTRmir-1bs->NotI) V	This Study	MLC1802
Caenorhabditis elegans: luc132(vha-1::3'UTRmir-1bs->NotI) III; luc130 (vha-11::3'UTRmir-1bs->NotI) IV; luc135(vha-8::3'UTRmir-1bs->NotI)IV; luc133(vha-13::3'UTRmir-1bs->NotI) V	This Study	MLC1804
Caenorhabditis elegans: luc138(vha-14::3'UTRmir-1bs->NotI) III; luc132(vha-1::3'UTRmir-1bs->NotI) III; luc130 (vha-11::3'UTRmir-1bs->NotI) IV; luc135(vha-8::3'UTRmir-1bs->NotI) IV; luc133(vha-13::3'UTRmir-1bs->NotI) V	This Study	MLC1834
Caenorhabditis elegans: luc139(vha-12::3'UTRmir-1bs->NotI,BamHI, EcoRI) X; luc138(vha-14::3'UTRmir-1bs->NotI) III; luc132(vha-1::3'UTRmir-1bs->NotI) III; luc130 (vha-11::3'UTRmir-1bs->NotI) IV; luc135(vha-8::3'UTRmir-1bs->NotI) IV; luc133(vha-13::3'UTRmir-1bs->NotI). Referred as 6x-vha^{NotI}	This Study	MLC1843
Caenorhabditis elegans: zcIs14 [myo-3p::GFP(mit)]; luc132(vha-1::3'UTRmir-1bs->NotI) III; luc138(vha-14::3'UTRmir-1bs->NotI) III; luc130 (vha-11::3'UTRmir-1bs->NotI) IV; luc135(vha-8::3'UTRmir-1bs->NotI) IV; luc133(vha-13::3'UTRmir-1bs->NotI) V; luc139(vha-12::3'UTRmir-1bs->NotI,BamHI, EcoRI) X	This Study	MLC1845
Caenorhabditis elegans: luc132(vha-1::3'UTRmir-1bs->NotI) III; luc138(vha-14::3'UTRmir-1bs->NotI) III; luc130 (vha-11::3'UTRmir-1bs->NotI) IV; luc135(vha-8::3'UTRmir-1bs->NotI) IV; luc133(vha-13::3'UTRmir-1bs->NotI) V; luc139(vha-12::3'UTRmir-1bs->NotI,BamHI, EcoRI) X; rmIs133 [unc-54p::Q40::YFP] X	This Study	MLC2266
Caenorhabditis elegans: lucEx421 (mir-4813 1kb upstream p::myr::gfp::1kb unc-54 3'UTR downstream); luc132(vha-1::3'UTRmir-1bs->NotI) III; luc138(vha-14::3'UTRmir-1bs->NotI) III; luc130 (vha-11::3'UTRmir-1bs->NotI) IV; luc135(vha-8::3'UTRmir-1bs->NotI) IV; luc133(vha-13::3'UTRmir-1bs->NotI) V; luc139(vha-12::3'UTRmir-1bs->NotI,BamHI, EcoRI) X	This Study	MLC1847
Caenorhabditis elegans: luc145(dct-1::3'UTRmir-1bs(x2)->NotI) X	This Study	MLC1947
Caenorhabditis elegans: zcIs14 [myo-3p::GFP(mit)]; luc145(dct-1::3'UTRmir-1bs(x2)->NotI) X	This Study	MLC1987
Caenorhabditis elegans: rmIs133 [unc-54p::Q40::YFP] X; luc145(dct-1::3'UTRmir-1bs(x2)->NotI) X	This Study	MLC2267
Caenorhabditis elegans: vha-1(luc161)/hT2[bli-4(e937) let-?(q782) qIs48] III	This Study	MLC2230
Caenorhabditis elegans: vha-1(luc161)/hT2[bli-4(e937) let-?(q782) qIs48] III; rmIs133 [unc-54p::Q40::YFP] X	This Study	MLC2284
Caenorhabditis elegans: mir-1(luc108) I; vha-1(luc161)/hT2[bli-4(e937) let-?(q782) qIs48] III; rmIs133 [unc-54p::Q40::YFP] X	This Study	MLC2285
Caenorhabditis elegans: vha-2(ok619) III	Caenorhabditis Genetics Center	RB807
Caenorhabditis elegans: zcIs14 [myo-3p::GFP(mit)]; vha-2(ok619) III	This Study	MLC1835
Caenorhabditis elegans: vha-3(ok1501) IV	Caenorhabditis Genetics Center	VC1003
Caenorhabditis elegans: zcIs14 [myo-3p::GFP(mit)]; vha-3(ok1501) IV	This Study	MLC1837
Caenorhabditis elegans: vha-2(ok619) III; rmIs133 [unc-54p::Q40::YFP] X	This Study	MLC1839
Caenorhabditis elegans: vha-3(ok1501) IV; rmIs133 [unc-54p::Q40::YFP] X	This Study	MLC1840

Caenorhabditis elegans: vha-8(jh135)/bli-6(sc16) egl-19(ad695) unc-24(e318) IV	Caenorhabditis Genetics Center	MLC1842
Caenorhabditis elegans: vha-8(jh135)/tmC25 [unc-5(tmIs1241)] IV; rmIs133 [unc-54p::Q40::YFP] X	This study	MLC2078
Caenorhabditis elegans: vha-5(mc38)/tmC5 [F36H1.3(tmIs1220)] IV	Caenorhabditis Genetics Center	KJ487
Caenorhabditis elegans: zcIs14 [myo-3::GFP(mit)]; vha-5(mc38)/tmC5 [F36H1.3(tmIs1220)] IV	This Study	MLC2079
Caenorhabditis elegans: vha-5(mc38)/tmC5 [F36H1.3(tmIs1220)] IV; rmIs133 [unc-54p::Q40::YFP] X	This Study	MLC2081
Caenorhabditis elegans: lucEx1114(WRM065bD07, ttx-3p::mCherry)	This Study	MLC1955
Caenorhabditis elegans: lucEx1115(WRM065bD07, ttx-3p::mCherry)	This Study	MLC1956
Caenorhabditis elegans: lucEx1114(WRM065bD07, ttx-3p::mCherry); rmIs133 [unc-54p::Q40::YFP] X	This Study	MLC1999
Caenorhabditis elegans: lucEx1115(WRM065bD07, ttx-3p::mCherry); mir-1(luc108) I; rmIs133 [unc-54p::Q40::YFP] X	This Study	MLC2003
Caenorhabditis elegans: lucEx1114(WRM065bD07, ttx-3p::mCherry); mir-1(luc108) I; rmIs133 [unc-54p::Q40::YFP] X	This Study	MLC2004
Caenorhabditis elegans: lucEx1115(WRM065bD07, ttx-3p::mCherry); rmIs133 [unc-54p::Q40::YFP] X	This Study	MLC2005
Caenorhabditis elegans: luc130 (vha-11::3'UTRmir-1bs->NotI) IV; rmIs133 [unc-54p::Q40::YFP] X	This Study	MLC2149
Caenorhabditis elegans: luc132(vha-1::3'UTRmir-1bs->NotI) III; luc138(vha-14::3'UTRmir-1bs->NotI) III; rmIs133 [unc-54p::Q40::YFP] X	This Study	MLC2150
Caenorhabditis elegans: luc135(vha-8::3'UTRmir-1bs->NotI) IV; rmIs133 [unc-54p::Q40::YFP] X	This Study	MLC2151
Caenorhabditis elegans: luc139(vha-12::3'UTRmir-1bs->NotI,BamHI, EcoRI) X; rmIs133 [unc-54p::Q40::YFP] X	This Study	MLC2152
Caenorhabditis elegans: luc133(vha-13::3'UTRmir-1bs->NotI) V; rmIs133 [unc-54p::Q40::YFP] X	This Study	MLC2153
Caenorhabditis elegans: luc132(vha-1::3'UTRmir-1bs->NotI) III; rmIs133 [unc-54p::Q40::YFP] X	This Study	MLC2154
Caenorhabditis elegans: zcIs14 [myo-3p::GFP(mit)]; luc132(vha-1::3'UTRmir-1bs->NotI) III	This Study	MLC2214
Caenorhabditis elegans: zcIs14 [myo-3p::GFP(mit)]; luc133(vha-13::3'UTRmir-1bs->NotI) V	This Study	MLC2215
Caenorhabditis elegans: lucEx1115(WRM065bD07, ttx-3p::mCherry); zcIs14 [myo-3p::GFP(mit)]	This Study	MLC2228
Caenorhabditis elegans: lucEx1115(WRM065bD07, ttx-3p::mCherry); zcIs14 [myo-3p::GFP(mit)]; mir-1(luc108) I	This Study	MLC2218
Caenorhabditis elegans: zcIs14 [myo-3p::GFP(mit)]; luc130 (vha-11::3'UTRmir-1bs->NotI) IV	This Study	MLC2302
Caenorhabditis elegans: zcIs14 [myo-3p::GFP(mit)]; luc134(vha-14::3'UTRmir-1bs->NotI) III	This Study	MLC2303
Caenorhabditis elegans: luc179(tbc-7::3'UTRmir-1bs(x2)->NotI,BamHI) X	This Study	MLC2364
Caenorhabditis elegans: rmIs133 [unc-54p::Q40::YFP]; luc179(tbc-7::3'UTRmir-1bs(x2)->NotI,BamHI) X.	This Study	MLC2390
Caenorhabditis elegans: zcIs14 [myo-3::GFP(mit)]; luc179(tbc-7::3'UTRmir-1bs(x2)->NotI,BamHI) X	This Study	MLC2391
Caenorhabditis elegans: lucEx1207[myo-3p::YFP]	This Study	MLC2232
Caenorhabditis elegans: lucEx1207[myo-3p::YFP]; mir-1(luc108) I	This Study	MLC2277

Caenorhabditis elegans: lucEx421[mir-4813 1kb upstream p::myr::gfp::1kb unc-54 3'UTR downstream; ttx-3p::mCherry]; luc145(dct-1::3'UTRmir-1bs(x2)->NotI) X	This Study	MLC2399
Caenorhabditis elegans: lucEx1114[WRM065bD07, ttx-3p::mCherry]; rmIs133 [unc-54p::Q40::YFP]; luc145(dct-1::3'UTRmir-1bs(x2)->NotI) X	This Study	MLC2409
Caenorhabditis elegans: oxIs322 II; unc-119(ed3) III; lucEx1311(myo-3p::R2pH-LAMP1-3xFLAG::unc-54 3'UTR; ttx-3p::mCherry)	This Study	MLC2465
Caenorhabditis elegans: oxIs322 II; unc-119(ed3) III; lucEx1311(myo-3p::R2pH-LAMP1-3xFLAG::unc-54 3'UTR; ttx-3p::mCherry); mir-1(luc108) I	This Study	MLC2481
Caenorhabditis elegans: luc193(dct-1::3'UTRmir-1bs(x2)->NotI); zcIs14; luc139(vha-12::3'UTRmir-1bs->NotI,BamHI, EcoRI); luc138(vha-14::3'UTRmir-1bs->NotI); luc132(vha-1::3'UTRmir-1bs->NotI); luc130 (vha-11::3'UTRmir-1bs->NotI);luc133(vha-13::3'UTRmir-1bs->NotI); luc135(vha-8::3'UTRmir-1bs->NotI)	This Study	MLC2529
Caenorhabditis elegans: luc195(dct-1::3'UTRmir-1bs(x2)->NotI); rmIs133 [unc-54p::Q40::YFP]; luc139(vha-12::3'UTRmir-1bs->NotI,BamHI, EcoRI); luc138(vha-14::3'UTRmir-1bs->NotI); luc132(vha-1::3'UTRmir-1bs->NotI); luc130 (vha-11::3'UTRmir-1bs->NotI);luc133(vha-13::3'UTRmir-1bs->NotI); luc135(vha-8::3'UTRmir-1bs->NotI)	This Study	MLC2545
Caenorhabditis elegans: lucEx1353(WRM0637aE07(mir-1 fosmid); ttx-3p::mCherry); rmIs133 [unc-54p::Q40::YFP]; mir-1(luc108) I	This Study	MLC2526
Caenorhabditis elegans: lucEx1354(WRM0637aE07(mir-1 fosmid); ttx-3p::mCherry); rmIs133 [unc-54p::Q40::YFP]; mir-1(luc108) I	This Study	MLC2527
Caenorhabditis elegans: lucEx1355(WRM0637aE07(mir-1 fosmid); ttx-3p::mCherry); rmIs133 [unc-54p::Q40::YFP]; mir-1(luc108) I	This Study	MLC2528
Drosophila melanogaster: w Canton S	Bloomington Drosophila Stock Center	BDSC-64349
Drosophila melanogaster: w[*]; mir-1[KO]/CyO, P{w[+mC]=GAL4-twi.G}2.2, P{UAS-2xEGFP}AH2.2	Bloomington Drosophila Stock Center	BDSC-58879
Drosophila melanogaster: w[*]; Vha68-2[R6] P{ry[+t7.2]=neoFRT}40A/CyO, P{w[+mC]=GAL4-twi.G}2.2, P{UAS-2xEGFP}AH2.2	Bloomington Drosophila Stock Center	BDSC-39621
Drosophila melanogaster: y[1] w[*]; Mi{y[+mDint2]=MIC}Vha100-3[MI03166] sano[MI03166]	Bloomington Drosophila Stock Center	BDSC-36212
Drosophila melanogaster: y[1]; P{y[+mDint2] w[BR.E.BR]=SUPor-P}Vha44[KG00915]/CyO; ry[506]	Bloomington Drosophila Stock Center	BDSC-13299
Drosophila melanogaster: y[1] w[*]; P{w[+mC]=lacW}Vha55[j2E9]/TM3, Sb[1]	Bloomington Drosophila Stock Center	BDSC-12128
Drosophila melanogaster: w[*]; In(2LR)noc[4L]Sco[rv9R], b[1]/CyO, P{w[+mC]=ActGFP}JMR1	Bloomington Drosophila Stock Center	BDSC-4533
Drosophila melanogaster: w[*]; Sb[1]/TM3, P{w[+mC]=ActGFP}JMR2, Ser[1]	Bloomington Drosophila Stock Center	BDSC-4534
Drosophila melanogaster: w[1118]; DCTN1-p150[1]/TM3, P{w[+mC]=sChFP}3, Sb[1]	Bloomington Drosophila Stock Center	BDSC-35524
Drosophila melanogaster: mir-1[KO], Vha68-2[R6] P{ry[+t7.2]=neoFRT}40A/ CyO, P{w[+mC]=ActGFP}JMR1	This Study	PGP_11

Drosophila melanogaster: mir-1[KO], Mi{y[+mDint2]=MIC}Vha100-3[MI03166] sano[MI03166]/ CyO, P{w[+mC]=ActGFP}JMR1	This Study	PGP_10
Drosophila melanogaster: mir-1[KO] / CyO, P{w[+mC]=ActGFP}JMR1 ; P{lacW}Vha55j2E9 / TM3, P{w[+mC]=sChFP}3, Sb[1]	This Study	PGP_12
Drosophila melanogaster: mir-1[KO], P{SUPor-P}Vha44KG00915/ CyO, P{w[+mC]=GAL4-Kr.C}DC3, P{w[+mC]=UAS-GFP.S65T}DC7	This Study	PGP_15
Drosophila melanogaster: w[*]; P{w[+mW.hs]=GawB}how[24B]	Bloomington Drosophila Stock Center	BDSC-1767
Drosophila melanogaster: y[1] w[*]; P{w[+mC]=UAS-Vha100-1.H}7.3; P{ry[+t7.2]=neoFRT}82B Vha100-1[1]/TM3, Sb[1]	Bloomington Drosophila Stock Center	BDSC-39669
Drosophila melanogaster: y[1] w[*]; P{w[+mC]=UAS-GFP::lacZ.nls}15.3; Sb/TM3,Ser	Peter Duchek Austria	PD-6451 (modified from BDSC-6451)

Table S2. Alt-R® CRISPR-Cas9 crRNA sequences for Homology-directed genome editing used in this study.

Oligonucleotides	
Sequence (5'→3')	Source
crRNA targeting *mir-1*<br>#1: AAGAAGTATGTAGAACGGGG	IDT
crRNA targeting *mir-1*<br>#2: TATAGAGTAGAATTGAATCT	IDT
crRNA targeting *vha-1* 3'UTR<br>#1: TCGGTTTATTTGGAATGTGG	IDT
crRNA targeting *vha-1* 3'UTR<br>#2: AACGCCGATATCATCAGTGA	IDT
crRNA targeting *vha-8* 3'UTR<br>#1: TAACATCATGGAATGTAGTT	IDT
crRNA targeting *vha-8* 3'UTR<br>#2: AGTTGGGCGATGAAATTACG	IDT
crRNA targeting *vha-11* 3'UTR<br>#1: AAATCGGAGAGTGTGTGAGG	IDT
crRNA targeting *vha-11* 3'UTR<br>#2: TTGGTCAAATTTGACCGGAA	IDT
crRNA targeting *vha-12* 3'UTR<br>#1: ATGAAGAGACAAGATAAGGT	IDT
crRNA targeting *vha-12* 3'UTR<br>#2: GGATTTCCAGCTATCACGGG	IDT
crRNA targeting *vha-13* 3'UTR<br>#1: AAGGAGGCGAATTGGGTGAG	IDT
crRNA targeting *vha-13* 3'UTR<br>#2: GCGACCAGATACATTGTGTG	IDT
crRNA targeting *vha-14* 3'UTR<br>#1: ATAGAAAACAGGAAGAACAT	IDT
crRNA targeting *vha-14* 3'UTR<br>#2: AAGTTTTAAAAGAAAATGGT	IDT
crRNA targeting *dct-1* 3'UTR<br>#1: GTACAGTGAAATGAGGTAAG	IDT
crRNA targeting *dct-1* 3'UTR<br>#2: CGCGATTTGCTCTCACGATT	IDT
crRNA targeting *tbc-7* 3'UTR<br>#1: ACGAGGAATTGACTAGAAGT	IDT
crRNA targeting *tbc-7* 3'UTR<br>#2: AAACATTGGAGGCGAGGAGT	IDT
crRNA targeting *vha-1*<br>#1: AGCAGCAGCAAATGCCATGG	IDT
crRNA targeting *vha-1*<br>#2: CATGCGCGGTTGTTGTGAGA	IDT

Table S3. Oligonucleotides sequences for qPCR used in this study.

Oligonucleotides	
Sequence (5'→3')	Gene
FW: CGACCCGATCTCGTTATGAAGGC	*Caenorhabditis elegans vha-1*
RV: GCGCGGTTGTTGTGAGAGG	*Caenorhabditis elegans vha-1*
FW: GCCGTGAGAAGGATCTTCGTC	*Caenorhabditis elegans vha-8*
RV: GACTTGTGGGACAATCTGGTTAGC	*Caenorhabditis elegans vha-8*
FW: GGAGACTCTTAAAGCAGGAAGAACG	*Caenorhabditis elegans vha-11*
RV: GGACCAGCGGCGGATCC	*Caenorhabditis elegans vha-11*
FW: CGTCGATCGTCAGCTTCACAATC	*Caenorhabditis elegans vha-12*
RV: CTGTTTTCGTAGTGACCTTGGGTG	*Caenorhabditis elegans vha-12*
FW: CTTCGTGAGATTTCTGGACGTCTC	*Caenorhabditis elegans vha-13*
RV: GGGTGGCGGATGTGACTG	*Caenorhabditis elegans vha-13*
FW: CTCGTCGAGCTCGCCACTC	*Caenorhabditis elegans vha-14*
RV: GTCCTGGTCCTTCCAATGCTTTTTG	*Caenorhabditis elegans vha-14*
FW: GAAAATACACCTCCAAAGACTGTCCG	*Caenorhabditis elegans dct-1*
RV: GATGTTTGATTAGTTTCCGGCAAACAG	*Caenorhabditis elegans dct-1*
FW: GAAAGAGAGGACTCGTTCAAAATATTGGG	*Caenorhabditis elegans tbc-7*
RV: CGCTGGAAGTAACATGGAACACC	*Caenorhabditis elegans tbc-7*
FW: TGGGTGCCTGAAATTTCGC	*Caenorhabditis elegans cdc-42*
RV: CTTCTCCTGTTGTGGTGGG	*Caenorhabditis elegans cdc-42*
FW: CAAGAGCGTGCCGACGCC	*Caenorhabditis elegans myo-2*
RV: GTTAGCTTGCTCCTCAGCTTCCT	*Caenorhabditis elegans myo-2*
FW: CTCGGGAACGCGGTCGAT	*Caenorhabditis elegans hlh-1*
RV: CCCCATCAATCACATCATTTGCTG	*Caenorhabditis elegans hlh-1*
FW: TCTGAGATCGTGCAACTGGTCG	*Drosophila melanogaster Vha68-2*
RV: ACGGGGTCCTTGAACTTCATGG	*Drosophila melanogaster Vha68-2*
FW: TCTACCCACCAGTCAACGTGC	*Drosophila melanogaster Vha55*
RV: GTTCTCATAGTTGCCCTGCGAG	*Drosophila melanogaster Vha55*
FW: ATCTGATCGAGAAGCCGGAGG	*Drosophila melanogaster VhaSFD*
RV: GCGACGGAAAGGATGATGGC	*Drosophila melanogaster VhaSFD*
FW: TCATGGCTAATGACTGGCTGACC	*Drosophila melanogaster Vha44*
RV: AACGAACCAAAGGACCAAACTGC	*Drosophila melanogaster Vha44*
FW: CGTCTGAAGGTGCTGAAAGTGC	*Drosophila melanogaster Vha26*
RV: GCGTATTGGGCACCTTGATGC	*Drosophila melanogaster Vha26*
FW: TCGCCCTGGTAGGAATAGCC	*Drosophila melanogaster Vha100-3*
RV: GGCAGAGTGTTGGCGAATCC	*Drosophila melanogaster Vha100-3*
FW: CTGGCTTTTGAGGCCGATCG	*Drosophila melanogaster VhaAC39-1*
RV: TGTTACGGCACTCCTGCTCC	*Drosophila melanogaster VhaAC39-1*
FW: TTGTGCTAAGTGTGTGCAGCG	*Drosophila melanogaster Actin42a*
RV: ATTACGCCCTGGTGACGTGG	*Drosophila melanogaster Actin42a*
FW: CCGGCGATTCCAAGAACAATCC	*Drosophila melanogaster* $EF1\alpha2$
RV: AACTCCTGGAAGCTCTCTACGC	*Drosophila melanogaster* $EF1\alpha2$

Table S4. Cq qPCR values corresponding to Figure S3C, F and Figure S6A.

			Cq value									Relative expression (%)		
			Biological replicate (BR) 1			Biological replicate (BR) 2			Biological replicate (BR) 3					
**Gene**	**Genotype**	**Nutritional state**	Technical replicate 1	Technical replicate 2	Technical replicate 3	Technical replicate 1	Technical replicate 2	Technical replicate 3	Technical replicate 1	Technical replicate 2	Technical replicate 3	BR 1	BR 2	BR 3
dct-1	wt	Fed	32.31	32.25	32.34	32.68	32.09	32.20	32.58	32.71	32.40	94.56	114.44	91.00
tbc-7	wt	Fed	31.03	30.90	30.88	31.09	31.25	31.13	30.70	30.88	31.01	91.77	96.75	111.48
cdc-42	wt	Fed	28.33	27.94	27.79	28.36	28.29	28.31	28.38	28.13	28.18	-	-	-
dct-1	*mir-1(luc108)*	Fed	31.15	31.11	30.65	31.14	31.29	31.05	31.70	32.24	31.53	312.98	273.85	200.45
tbc-7	*mir-1(luc108)*	Fed	30.43	30.32	30.53	30.12	30.29	30.32	30.68	30.69	30.39	172.42	195.01	178.32
cdc-42	*mir-1(luc108)*	Fed	28.43	28.57	28.26	28.32	28.38	28.55	28.75	28.55	28.59	-	-	-
vha-1	wt	Fed	26.66	26.30	26.44	27.12	27.09	27.14	26.72	26.77	26.86	119.50	93.13	87.37
vha-8	wt	Fed	25.83	25.63	25.58	26.19	26.08	26.09	25.82	25.87	26.00	111.72	100.98	87.30
vha-11	wt	Fed	26.66	26.39	26.13	26.84	27.22	27.16	26.82	26.63	26.68	120.21	91.95	87.84
cdc-42	wt	Fed	28.80	28.51	28.83	29.13	28.90	28.98	28.52	28.53	28.67	-	-	-
vha-1	*mir-1(luc108)*	Fed	26.47	26.39	26.33	26.35	26.45	26.31	26.52	26.65	26.38	160.49	159.54	181.57
vha-8	*mir-1(luc108)*	Fed	25.90	25.84	25.69	25.68	25.67	26.04	25.87	25.92	25.86	131.05	128.65	152.87
vha-11	*mir-1(luc108)*	Fed	26.74	26.53	26.51	26.51	26.65	26.50	27.00	27.22	26.73	134.19	134.60	125.76
cdc-42	*mir-1(luc108)*	Fed	29.19	28.95	29.06	28.99	28.94	29.18	29.53	29.37	29.21	-	-	-
vha-12	wt	Fed	29.24	29.46	29.47	29.27	29.51	29.65	29.49	30.43	29.36	82.05	121.42	96.53
vha-13	wt	Fed	25.74	25.56	25.58	26.00	26.00	25.97	25.81	25.75	25.76	90.61	110.79	98.60
vha-14	wt	Fed	27.19	26.96	26.95	27.17	27.35	27.14	27.52	27.49	27.38	91.53	126.68	81.79
cdc-42	wt	Fed	28.16	28.17	28.16	28.85	28.90	28.71	28.27	28.18	28.86	-	-	-
vha-12	*mir-1(luc108)*	Fed	28.94	28.95	29.06	28.97	28.86	28.88	29.24	29.38	29.31	130.74	132.56	152.51
vha-13	*mir-1(luc108)*	Fed	25.57	25.53	25.66	25.24	25.39	25.37	25.48	25.50	25.46	111.68	127.90	176.29
vha-14	*mir-1(luc108)*	Fed	27.19	26.83	26.82	26.86	26.76	26.93	27.10	27.11	27.02	116.66	119.80	156.02
cdc-42	*mir-1(luc108)*	Fed	28.67	28.36	28.26	28.30	28.55	28.26	28.71	28.76	29.47	-	-	-

			Cq value									Relative expression (%)		
			Biological replicate (BR) 1			Biological replicate (BR) 2			Biological replicate (BR) 3					
**Gene**	**Genotype**	**Nutritional state**	Technical replicate 1	Technical replicate 2	Technical replicate 3	Technical replicate 1	Technical replicate 2	Technical replicate 3	Technical replicate 1	Technical replicate 2	Technical replicate 3	BR 1	BR 2	BR 3
dct-1	wt	Starved	30.26	30.11	30.41	30.83	30.47	30.54	30.25	30.49	30.33	79.16	102.49	118.36
cdc-42	wt	Starved	29.50	29.35	29.23	30.33	29.87	30.05	29.73	29.85	30.54	-	-	-
dct-1	*mir-1(luc108)*	Starved	30.00	29.93	29.78	30.54	30.30	30.51	30.51	30.36	30.28	259.20	187.46	205.33
cdc-42	*mir-1(luc108)*	Starved	30.83	30.72	30.60	30.93	30.66	30.80	30.84	32.50	30.88	-	-	-
tbc-7	wt	Starved	33.08	32.65	32.90	32.36	32.66	31.98	32.72	32.62	32.50	102.16	96.34	101.50
cdc-42	wt	Starved	30.42	30.07	30.13	29.73	29.46	29.54	29.86	30.06	29.87	-	-	-
tbc-7	*mir-1(luc108)*	Starved	32.58	32.14	32.39	31.35	31.88	31.62	32.16	31.73	31.90	159.13	188.04	129.45
cdc-42	*mir-1(luc108)*	Starved	30.54	30.21	30.27	29.93	29.75	29.80	29.74	29.64	29.42	-	-	-
vha-1	wt	Starved	28.18	27.83	27.89	28.41	28.41	28.51	28.17	28.15	28.07	92.74	91.16	116.10
vha-8	wt	Starved	27.64	27.71	27.39	28.10	28.09	27.89	27.73	27.76	27.71	91.91	92.12	115.97
vha-11	wt	Starved	28.40	28.13	28.05	28.97	28.83	28.85	28.63	28.65	28.75	105.23	89.15	105.62
cdc-42	wt	Starved	29.83	29.41	29.24	30.22	29.89	29.72	29.84	30.24	29.86	-	-	-
vha-1	*mir-1(luc108)*	Starved	28.19	28.05	27.88	28.05	28.06	28.10	28.35	28.31	28.31	165.58	158.96	143.19
vha-8	*mir-1(luc108)*	Starved	27.97	27.49	27.58	27.82	27.78	27.63	28.00	27.98	27.96	160.75	151.03	137.49
vha-11	*mir-1(luc108)*	Starved	28.89	28.59	28.84	28.88	28.95	28.77	29.35	29.08	29.04	131.88	121.50	106.37
cdc-42	*mir-1(luc108)*	Starved	30.71	30.21	30.28	30.55	30.13	30.43	30.71	30.39	30.32	-	-	-
vha-12	wt	Starved	30.62	30.71	30.25	30.53	30.61	30.83	31.07	31.03	30.92	99.95	115.64	84.41
vha-13	wt	Starved	28.16	28.04	27.85	28.27	28.28	28.28	28.20	28.22	28.28	97.55	103.21	99.23
vha-14	wt	Starved	28.73	28.29	28.31	29.17	29.15	29.18	28.42	28.24	28.39	99.25	76.08	124.67
cdc-42	wt	Starved	29.22	29.29	28.98	29.42	29.38	29.70	29.47	29.55	29.18	-	-	-
vha-12	*mir-1(luc108)*	Starved	30.52	30.50	30.21	30.57	30.08	30.18	30.54	30.45	30.98	155.90	197.11	178.30
vha-13	*mir-1(luc108)*	Starved	27.69	27.52	27.36	27.68	27.67	27.67	28.04	27.97	27.75	198.00	206.11	204.48
vha-14	*mir-1(luc108)*	Starved	28.79	28.51	28.44	28.54	28.62	28.71	29.13	29.11	29.06	129.91	145.54	123.17
cdc-42	*mir-1(luc108)*	Starved	29.87	29.55	29.64	30.18	29.70	29.80	30.41	29.84	30.14	-	-	-
hlh-1	wt	Starved	33.63	33.51	34.03	33.35	33.62	33.26	32.89	33.17	33.63	94.60	106.17	99.24
cdc-42	wt	Starved	29.80	29.54	29.42	29.47	29.40	29.45	29.19	29.06	29.24	-	-	-
hlh-1	*mir-1(luc108)*	Starved	33.42	33.45	33.02	33.40	33.62	33.89	33.77	33.61	33.13	203.10	108.03	130.50
cdc-42	*mir-1(luc108)*	Starved	30.40	30.41	29.97	29.65	29.59	29.83	29.87	29.88	29.73	-	-	-
myo-2	wt	Starved	28.18	28.39	28.58	29.03	29.09	29.38	29.29	29.41	29.63	120.19	94.70	85.11
cdc-42	wt	Starved	27.71	27.33	27.30	27.81	28.01	27.85	28.07	28.05	27.91	-	-	-
myo-2	*mir-1(luc108)*	Starved	31.29	30.59	30.33	32.15	32.20	32.95	29.70	29.95	29.76	36.58	18.63	55.00
cdc-42	*mir-1(luc108)*	Starved	28.33	28.11	27.80	28.87	28.82	28.74	27.94	27.81	27.48	-	-	-

			Cq value									Relative expression (%)		
			Biological replicate (BR) 1			Biological replicate (BR) 2			Biological replicate (BR) 3					
**Gene**	**Genotype**	**Nutritional state**	Technical replicate 1	Technical replicate 2	Technical replicate 3	Technical replicate 1	Technical replicate 2	Technical replicate 3	Technical replicate 1	Technical replicate 2	Technical replicate 3	BR 1	BR 2	BR 3
dct-1	wt	Fed	31.75	31.35	31.41	32.09	32.17	32.07	32.25	32.17	32.72	162.86	64.70	72.45
tbc-7	wt	Fed	31.59	31.56	31.31	30.85	30.43	30.67	30.57	30.50	30.69	83.21	89.86	126.93
cdc-42	wt	Fed	28.96	28.56	28.37	28.01	27.92	27.78	28.46	28.31	28.26	-	-	-
dct-1	*6x-vha*^{NotI}	Fed	32.34	32.28	31.78	31.94	31.61	31.94	31.03	31.28	31.01	116.96	113.44	198.04
tbc-7	*6x-vha*^{NotI}	Fed	30.82	30.87	30.60	30.20	30.20	30.33	30.30	30.58	30.47	152.87	171.80	157.72
cdc-42	*6x-vha*^{NotI}	Fed	28.82	28.86	28.68	28.36	28.60	28.36	28.47	28.60	28.48	-	-	-
vha-1	wt	Fed	27.22	26.78	26.86	26.39	26.38	26.89	26.86	26.81	26.95	118.42	90.11	91.47
vha-8	wt	Fed	26.30	26.06	26.16	25.68	25.65	25.54	26.06	26.07	26.06	113.95	96.28	89.78
vha-11	wt	Fed	27.17	27.05	26.81	26.19	26.21	26.22	26.74	26.75	26.78	104.29	104.98	90.73
cdc-42	wt	Fed	29.29	29.04	28.98	28.32	28.32	28.30	28.57	28.82	28.56	-	-	-
vha-1	*6x-vha*^{NotI}	Fed	27.45	27.28	27.13	26.85	26.94	27.07	27.20	27.24	27.10	101.19	102.46	98.85
vha-8	*6x-vha*^{NotI}	Fed	26.08	25.95	25.64	25.53	25.52	25.85	25.96	25.90	25.79	149.34	143.95	136.49
vha-11	*6x-vha*^{NotI}	Fed	26.64	26.51	26.55	26.43	26.48	26.55	26.85	26.65	26.66	152.68	129.71	124.71
cdc-42	*6x-vha*^{NotI}	Fed	29.39	29.19	29.06	28.84	28.78	29.08	29.25	28.94	29.02	-	-	-
vha-12	wt	Fed	29.91	29.51	29.37	28.99	29.08	28.94	29.30	29.26	30.48	96.01	90.75	113.25
vha-13	wt	Fed	26.14	26.14	26.05	25.36	25.62	25.33	25.62	25.56	25.97	92.62	92.33	115.05
vha-14	wt	Fed	27.42	27.35	27.15	26.65	26.80	26.62	27.22	27.28	27.41	103.25	98.98	97.77
cdc-42	wt	Fed	28.70	28.75	28.55	28.09	27.99	27.89	28.50	28.38	28.87	-	-	-
vha-12	*6x-vha*^{NotI}	Fed	29.04	29.13	29.09	28.93	29.00	29.04	29.29	29.35	29.16	133.22	128.49	101.98
vha-13	*6x-vha*^{NotI}	Fed	25.46	25.42	25.39	25.33	25.28	25.22	25.52	25.35	25.53	145.23	144.89	121.95
vha-14	*6x-vha*^{NotI}	Fed	27.23	27.23	27.20	27.13	27.19	27.21	27.46	27.38	27.38	106.93	99.13	81.37
cdc-42	*6x-vha*^{NotI}	Fed	28.75	28.66	28.47	28.43	28.39	28.62	28.44	28.42	28.41	-	-	-
dct-1	wt	Fed	31.39	31.05	31.29	31.95	31.84	32.13	33.06	33.12	33.54	173.00	94.98	32.02
tbc-7	wt	Fed	31.02	31.01	30.84	31.36	31.24	31.36	30.57	30.92	30.75	116.87	83.05	100.08
vha-8	wt	Fed	25.31	25.10	24.97	25.23	25.30	25.20	25.07	24.92	24.94	112.49	94.91	92.60
cdc-42	wt	Fed	29.16	28.66	28.41	28.53	28.62	28.69	28.32	28.30	28.31	-	-	-
dct-1	*dct-1*^{NotI}	Fed	31.35	31.04	31.03	30.83	30.69	31.09	31.16	31.12	31.11	148.19	271.37	124.17
tbc-7	*dct-1*^{NotI}	Fed	30.41	30.27	30.12	30.92	30.98	31.10	30.08	30.02	29.80	150.82	137.70	153.95
vha-8	*dct-1*^{NotI}	Fed	24.88	24.64	24.69	25.33	25.23	25.29	24.71	24.55	24.46	117.99	122.57	109.64
cdc-42	*dct-1*^{NotI}	Fed	28.51	28.43	28.32	28.91	28.98	29.17	28.34	28.15	27.96	-	-	-

			Cq value									Relative expression (%)		
			Biological replicate (BR) 1			Biological replicate (BR) 2			Biological replicate (BR) 3					
**Gene**	**Genotype**	**Nutritional state**	Technical replicate 1	Technical replicate 2	Technical replicate 3	Technical replicate 1	Technical replicate 2	Technical replicate 3	Technical replicate 1	Technical replicate 2	Technical replicate 3	BR 1	BR 2	BR 3
dct-1	wt	Starved	30.51	30.38	30.29	30.68	30.84	30.74	30.48	30.38	-	123.67	78.20	98.13
cdc-42	wt	Starved	29.71	29.61	29.67	29.48	29.32	29.30	29.20	29.94	28.96	-	-	
dct-1	*6x-vha*^{NotI}	Starved	29.71	29.84	29.77	29.74	29.69	29.66	29.42	29.46	29.32	116.17	90.88	90.55
cdc-42	*6x-vha*^{NotI}	Starved	29.10	28.93	28.84	28.61	28.53	28.44	28.28	28.24	28.16	-	-	-
vha-1	wt	Starved	27.82	27.73	27.54	27.97	28.04	27.52	-	-	-	109.76	90.24	-
vha-8	wt	Starved	27.25	26.94	26.60	26.95	26.77	26.51	-	-	-	98.28	101.72	-
vha-11	wt	Starved	28.28	28.11	27.98	28.17	28.09	27.77	-	-	-	100.83	99.17	-
cdc-42	wt	Starved	29.01	28.81	28.17	28.77	28.53	28.28	-	-	-	-	-	-
vha-1	*6x-vha*^{NotI}	Starved	27.42	27.31	27.07	27.78	27.65	27.55	27.27	27.30	27.14	135.74	133.95	120.63
vha-8	*6x-vha*^{NotI}	Starved	26.21	25.92	25.47	26.54	26.47	26.33	25.86	25.65	25.55	188.58	163.05	186.29
vha-11	*6x-vha*^{NotI}	Starved	27.33	27.05	26.87	28.18	27.94	27.60	27.17	27.37	27.02	189.96	139.17	153.56
cdc-42	*6x-vha*^{NotI}	Starved	28.77	28.59	28.26	29.07	29.07	28.59	28.41	28.50	28.10	-	-	-
vha-12	wt	Starved	29.30	29.15	29.29	29.46	29.26	29.00	-	-	-	100.44	99.56	-
vha-13	wt	Starved	27.30	27.12	26.97	27.28	27.09	26.82	-	-	-	98.38	101.62	-
vha-14	wt	Starved	28.17	28.02	27.50	27.89	28.07	27.31	-	-	-	95.98	104.02	-
cdc-42	wt	Starved	28.71	28.40	28.18	28.47	28.49	28.28	-	-	-	-	-	-
vha-12	*6x-vha*^{NotI}	Starved	28.42	28.03	28.18	29.15	29.10	29.14	28.51	28.47	28.45	195.75	151.28	156.32
vha-13	*6x-vha*^{NotI}	Starved	26.31	26.12	25.83	26.66	26.53	26.32	25.93	25.71	25.76	192.01	210.73	225.60
vha-14	*6x-vha*^{NotI}	Starved	27.57	27.21	27.03	28.02	27.99	27.74	27.31	27.28	27.10	140.36	131.33	138.78
cdc-42	*6x-vha*^{NotI}	Starved	28.52	28.36	28.19	29.20	29.07	28.45	28.42	28.41	28.08	-	-	-
dct-1	wt	Starved	29.92	29.52	29.72	28.50	28.36	28.43	29.88	29.95	30.00	115.21	106.67	78.12
tbc-7	wt	Starved	31.58	31.66	31.69	30.39	30.48	30.35	31.73	31.63	31.83	113.65	101.42	84.93
vha-12	wt	Starved	30.14	30.02	29.93	28.58	28.47	28.45	29.61	29.71	29.74	96.89	105.74	97.37
cdc-42	wt	Starved	29.53	29.22	29.13	27.85	28.04	27.79	29.08	28.76	29.04	-	-	-
dct-1	*dct-1*^{NotI}	Starved	30.05	29.74	29.60	29.06	29.13	29.09	29.14	29.11	29.05	128.85	126.70	96.54
tbc-7	*dct-1*^{NotI}	Starved	31.81	31.94	31.57	30.81	31.02	31.19	30.47	30.76	30.50	122.20	125.87	129.58
vha-12	*dct-1*^{NotI}	Starved	30.29	30.01	30.00	29.11	29.09	29.34	29.24	29.38	29.32	108.87	124.21	86.85
cdc-42	*dct-1*^{NotI}	Starved	29.64	29.66	29.31	28.72	28.61	29.09	28.52	28.49	28.25	-	-	-
vha-1	wt	Starved	27.95	27.94	27.64	26.44	26.55	26.46	27.61	27.55	27.43	100.75	101.48	97.76
cdc-42	wt	Starved	29.62	29.23	29.17	27.95	27.99	28.02	29.09	28.87	28.99	-	-	-
vha-1	*dct-1*^{NotI}	Starved	28.08	27.96	27.90	27.16	27.18	27.14	27.16	26.94	26.84	108.73	103.49	103.84
cdc-42	*dct-1*^{NotI}	Starved	29.63	29.40	29.72	28.74	28.58	28.76	28.61	28.57	28.37	-	-	-

			Cq value									Relative expression (%)		
			Biological replicate (BR) 1			Biological replicate (BR) 2			Biological replicate (BR) 3					
**Gene**	**Genotype**	**Nutritional state**	Technical replicate 1	Technical replicate 2	Technical replicate 3	Technical replicate 1	Technical replicate 2	Technical replicate 3	Technical replicate 1	Technical replicate 2	Technical replicate 3	BR 1	BR 2	BR 3
dct-1	wt	Fed	31.30	31.10	30.81	30.97	31.11	30.92	31.16	31.18	31.35	124.29	88.45	87.26
tbc-7	wt	Fed	32.94	32.51	32.53	32.07	32.25	32.33	31.89	32.00	32.36	97.17	89.47	113.36
vha-8	wt	Fed	28.37	28.19	28.03	27.96	28.01	27.95	28.04	28.15	28.05	113.45	89.88	96.66
cdc-42	wt	Fed	30.27	30.06	29.94	29.60	29.62	29.38	29.76	29.79	29.68	-	-	-
dct-1	*tbc-7*^{NotI}	Fed	29.80	29.53	29.46	30.73	31.02	30.86	30.32	30.11	30.28	181.39	178.67	153.59
tbc-7	*tbc-7*^{NotI}	Fed	31.24	31.02	30.83	32.08	31.93	31.82	31.16	31.15	31.17	157.91	200.09	190.46
vha-8	*tbc-7*^{NotI}	Fed	27.62	27.51	27.59	28.44	28.62	28.64	28.22	28.09	28.14	91.83	110.18	81.37
cdc-42	*tbc-7*^{NotI}	Fed	29.08	29.17	29.24	30.49	30.32	30.45	29.68	29.42	29.59	-	-	-
dct-1	wt	Starved	31.30	31.10	30.81	30.97	31.11	30.92	31.16	31.18	31.35	124.29	88.45	87.26
tbc-7	wt	Starved	32.94	32.51	32.53	32.07	32.25	32.33	31.89	32.00	32.36	97.17	89.47	113.36
vha-1	wt	Starved	28.37	28.19	28.03	27.96	28.01	27.95	28.04	28.15	28.05	113.45	89.88	96.66
cdc-42	wt	Starved	30.27	30.06	29.94	29.60	29.62	29.38	29.76	29.79	29.68	-	-	-
dct-1	*tbc-7*^{NotI}	Starved	29.80	29.53	29.46	30.73	31.02	30.86	30.32	30.11	30.28	181.39	178.67	153.59
tbc-7	*tbc-7*^{NotI}	Starved	31.24	31.02	30.83	32.08	31.93	31.82	31.16	31.15	31.17	157.91	200.09	190.46
vha-1	*tbc-7*^{NotI}	Starved	27.62	27.51	27.59	28.44	28.62	28.64	28.22	28.09	28.14	91.83	110.18	81.37
cdc-42	*tbc-7*^{NotI}	Starved	29.08	29.17	29.24	30.49	30.32	30.45	29.68	29.42	29.59	-	-	-

		Cq value									Relative expression (%)		
		**Biological replicate (BR) 1**			**Biological replicate (BR) 2**			**Biological replicate (BR) 3**					
**Gene**	**Genotype**	Technical replicate 1	Technical replicate 2	Technical replicate 3	Technical replicate 1	Technical replicate 2	Technical replicate 3	Technical replicate 1	Technical replicate 2	Technical replicate 3	BR 1	BR 2	BR 3
Vha68-2	wt	20,70	20,64	20,69	20,77	20,65	20,69	20,27	20,22	20,33	91.63	90.02	121.22
Vha55	wt	21,69	21,64	21,60	21,55	21,40	21,37	21,31	21,09	21,05	85.13	97.95	119.94
VhaSFD	wt	22,66	22,69	22,66	22,92	22,79	22,77	22,19	22,17	22,09	92.02	82.54	131.67
Vha26	wt	21,41	21,35	21,35	22,00	21,91	21,69	21,15	21,11	21,06	105.54	74.81	126.66
Vha44	wt	22,36	22,24	22,25	22,30	22,01	22,02	21,89	21,70	21,86	86.23	97.35	119.13
Vha100-3	wt	32,29	32,35	32,19	31,36	31,56	32,10	31,89	31,57	32,07	78.74	119.4	106.36
VhaAC39-1	wt	21,48	21,56	21,85	21,54	21,46	21,51	21,15	21,06	21,13	86.06	94.12	123.45
Actin42A	wt	25,97	25,61	25,91	25,33	24,96	24,98	25,04	24,68	24,77	-	-	-
EF1a2	wt	27,15	27,18	27,21	27,00	26,88	26,99	26,63	26,78	26,77	85.62	99.75	117.10
Vha68-2	*Dmir-1*^{KO}	20,50	20,44	20,54	20,53	20,34	20,36	20,16	20,08	20,21	141.97	139.80	180.51
Vha55	*Dmir-1*^{KO}	21,63	21,43	21,21	21,31	21,09	21,06	20,78	20,65	20,61	136.25	164.31	228.09
VhaSFD	*Dmir-1*^{KO}	22,39	22,45	22,37	22,24	22,07	22,08	22,17	22,01	22,07	152.10	184.37	190.41
Vha26	*Dmir-1*^{KO}	21,87	22,01	21,81	21,32	21,21	21,22	21,03	21,09	20,79	100.67	157.90	191.80
Vha44	*Dmir-1*^{KO}	21,93	21,93	21,83	21,92	21,75	21,68	21,45	21,40	21,41	155.61	168.06	215.94
Vha100-3	*Dmir-1*^{KO}	32,61	32,22	33,56	32,45	32,27	32,31	32,08	31,64	31,77	75.38	103.31	147.13
VhaAC39-1	*Dmir-1*^{KO}	21,48	21,43	21,38	21,41	21,29	21,34	21,04	20,86	21,22	136.31	144.40	177.91
Actin42A	*Dmir-1*^{KO}	26,17	25,86	25,80	26,13	25,44	25,71	25,44	25,07	25,75	-	-	-
EF1a2	*Dmir-1*^{KO}	28,01	27,86	27,67	27,97	27,80	27,69	27,37	27,15	27,22	74.05	75.77	112.46
